# Green synthesis of metallic nanoparticles using *Pistacia* species: improved stability and biological activities

**DOI:** 10.1039/d5na00644a

**Published:** 2025-07-24

**Authors:** Obaydah Abd Alkader Alabrahim, Ahmed Maher Abdeldayem, Hassan Mohamed El-Said Azzazy

**Affiliations:** a Department of Chemistry, School of Sciences & Engineering, The American University in Cairo AUC Avenue, SSE # 1184, P. O. Box 74 New Cairo 11835 Egypt Obaydah.alabrahim@aucegypt.edu hazzazy@aucegypt.edu; b Department of Nanobiophotonics, Leibniz Institute of Photonic Technology Albert Einstein Str. 9 Jena 07745 Germany

## Abstract

Plant extracts, rich in bioactive compounds such as phenolics and flavonoids, serve as reducing and stabilizing agents in nanoparticles fabrication. The *Pistacia* genus, comprising species like *P. atlantica*, *P. lentiscus*, and *P. vera*, has demonstrated significant potential in synthesizing metallic nanoparticles with enhanced physicochemical and biomedical properties. This review provides a comprehensive description of the phytochemical composition of different *Pistacia* species and their utilization in nanoparticles synthesis. It further explores the biological mechanisms underlying their antimicrobial, antioxidant, anti-inflammatory, and anticancer activities, along with their safety and stability profiles. The review highlights the promise of *Pistacia*-derived metallic nanoparticles in biomedical applications, while emphasizing mechanistic, toxicological, and stability considerations crucial for clinical translation.

## Abstract 2

This review highlights the promise of *Pistacia*-derived metallic nanoparticles in biomedical applications, while emphasizing mechanistic, toxicological, and stability considerations crucial for clinical translation.

## Graphical abstract

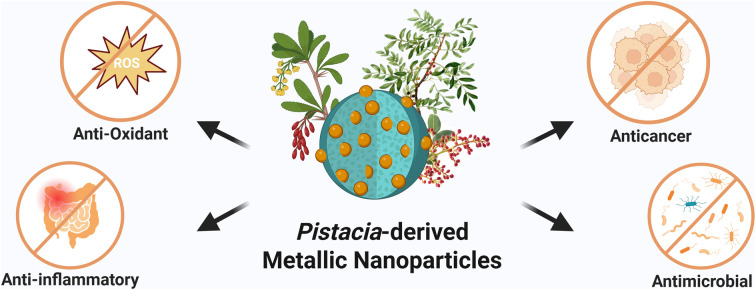

## 1. Introduction

The unique optical and physiochemical properties of nanomaterials have enabled major technological advances in chemistry, engineering, biotechnology, agriculture, medicine, drug delivery, and physics, attributed to unique features at the nanoscale.^1–6^ The fabrication of metal nanoparticles can be performed employing biological, physical, and chemical methods.^7^ These conventional methods are often associated with several drawbacks such as toxic byproducts, high pressure and temperature, and use of hazardous substances and toxic solvents.^8,9^ Hence, safer, eco-friendly, and cost-effective alternative methods for the fabrication of metallic nanoparticles, mainly using plant extracts, were introduced particularly for applications conducted in biomedical and pharmaceutical fields.^10,11^ Bioactive phytochemical compounds serve as reducing, capping, and stabilizing agents to transform metal ions into their corresponding nanoparticulate forms.^12^

The emergence of green synthesis for metallic nanoparticles leverages biological and natural resources, such as plants,^13,14^ algae,^15,16^ and microorganisms,^17–19^ to fabricate metallic nanoparticles. Plant extracts, in particular, offer the benefit of rapidly reducing metal ions owing to their simplified processing as compared to conventional methods, which usually require longer time and higher pressure and/or temperature.^20,21^ The ability of the functional groups of phytochemicals in plant extracts to donate electrons to metal ions enabled rapid and instant reduction of metal ions to their corresponding metallic nanoparticles.^22,23^ Additionally, phytochemicals, such as terpenoids, phenols, alkaloids, flavonoids, amides, amines, pigments, and proteins, play a crucial role in capping and stabilizing the synthesized nanoparticles.^17,18,21,24^

*Pistacia* trees, belonging to the Anacardiaceae family, have been a part of Mediterranean, Middle Eastern, and Central Asian landscapes for centuries. Their leaves and resinous bark, in particular, were traditionally utilized for medicinal purposes in these regions.^25–27^*Pistacia* extracts are well-established for their therapeutic effects, including antimicrobial, anti-inflammatory, and radical-scavenging activities. These properties are primarily attributed to different bioactive phytochemicals, such as terpenoids, terpenes, phenolics, and flavonoids.^25,28–32^ Recent reports have further highlighted the promise of *Pistacia* extracts in targeting cancer cells, employing different mechanisms showing superior anti-proliferative and pro-apoptotic effects against various tumor cells.^5,33–43^

This review presents a comprehensive and inclusive account of all *Pistacia*-mediated green synthesis studies reported in the literature on metallic nanoparticle fabrication. It explores the phytochemical foundation, fabrication strategies, biological activities, and mechanistic as well as safety and stability insights into these systems, reflecting their growing importance in biomedical and pharmaceutical applications.

## 2. Chemical composition of the *Pistacia* genus

**Fig. 1:**
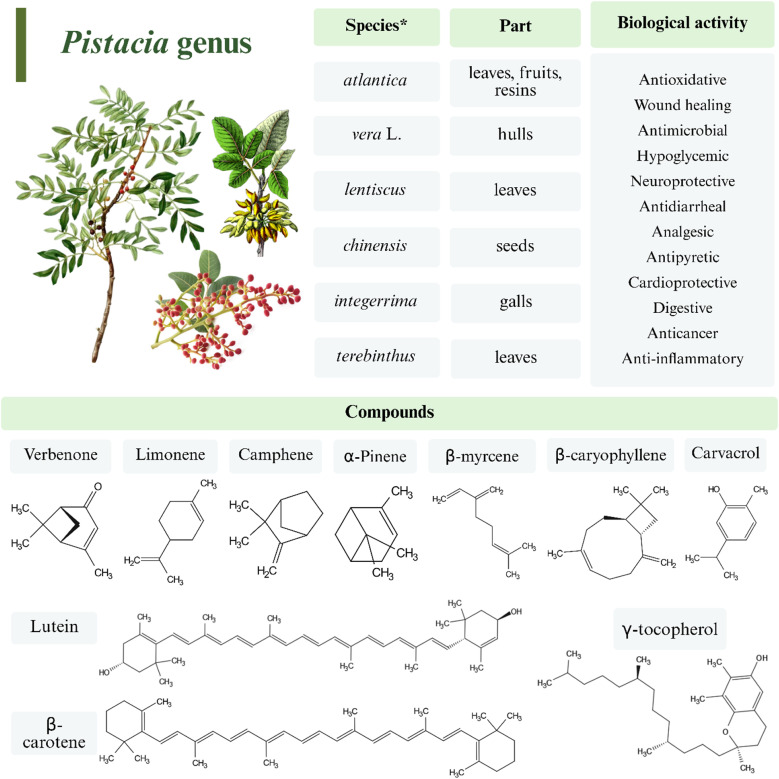
Schematic representation of the various parts of the *Pistacia* plant that can be isolated for bioactive compound extraction, along with their respective biological functions. * Given that the *Pistacia* genus comprises at least eleven valuable species, it should be noted that only the species used for the green synthesis of metallic nanoparticles are listed in this figure. The figure was drawn using BioRender.

The *Pistacia* genus, a rich source of bioactive phytochemicals, comprises a diverse array of compounds in resins, leaves, essential oils (EOs), and others. These compounds are isolated from multiple species demonstrating substantial biological activity, as shown in Fig. 1. As a prominent member of the Anacardiaceae family, these trees produce abundant resin from trunks and branches, highlighting their distinctive botanical traits.^25^

The composition of EOs derived from the *Pistacia* genus exhibits significant variation influenced by multiple factors, including species, tree gender, plant parts utilized (*e.g.*, leaves, fruits, galls, and resins), geographical origin, extraction solvents, methodologies, and other parameters.^44–46^ These oils are predominantly enriched with monoterpenes and oxygenated sesquiterpenes. Common constituents identified include myrcene, limonene, beta-gurjunene, germacrene, alpha-pinene, beta-pinene, muurolene, alpha-humulene, delta-3-carene, camphene, terpinen-4-ol, spathulenol, and *epi*-bicyclosesquiphellandrene, among others.^44–46^ Triterpenoids are considered as an important component of EOs derived from various *Pistacia* species, demonstrating significant anticancer potential. Among the triterpenic acids, 24*Z*-isomasticadienonic acid and 24*Z*-isomasticadienolic acid have been identified as key contributors to the pharmacological properties of resinous oils. Alpha-pinene emerges as a predominant constituent of EOs, alongside other notable compounds such as beta-myrcene, beta-pinene, carvacrol, camphene, limonene, beta-caryophyllene, verbenone, alpha-terpineol, and linalool.^47–50^ The common extraction method is hydrodistillation.^51^

Chemotypic classification *via* principal component analysis has revealed distinct clusters based on dominant components, including lutein, β-carotene, γ-tocopherol, and flavan-3-ols. Leaves of *P. lentiscus* contain germanicol, cadina-1,4-diene, and *trans*-squalene, while fresh unripe fruits yield higher EO quantities than leaves. Phenolics concentrate in nut skins, whereas lipophilic compounds dominate nutmeats, which are also rich in mono- and polyunsaturated fatty acids.^52–54^

Chios mastic gum, a resin of *P. lentiscus* var. *Chia*, exemplifies the genus pharmacological significance.^55,56^ This complex resin releases EOs with antimicrobial, anti-inflammatory, hypocholesterolemic, and anticancer activities. Historically utilized in Greek, Roman, and Arabian cultures, it serves both medicinal and culinary purposes.^56^ Recent studies underscore its potential in modulating immune responses and digestive health, reinforcing its value in nutraceutical and pharmaceutical applications.^55,57–59^

## 3. Green-synthesized metallic nanoparticles using *Pistacia* extracts

The green synthesis of metallic nanoparticles using *Pistacia* extracts typically involves the dual role of phytochemicals as both reducing and stabilizing agents. Phenolic compounds, flavonoids, tannins, and terpenoids found in these extracts donate electrons to reduce metal ions (*e.g.*, Ag^+^, Au^3+^, and Cu^2+^) to their zero-valent metallic states. Crucially, these and other functional groups (such as hydroxyl and carbonyl groups from various phytochemicals, including proteins and tannins) also stabilize the resulting nanoparticles by adsorbing onto their surface and preventing agglomeration. This biomolecular interaction governs the size, shape, and surface charge of the synthesized nanoparticles, influencing their final physicochemical and biological properties.

Green synthesis of metallic nanoparticles using *Pistacia* extracts typically involves aqueous extraction of different plant parts, followed by mixing with metal salt solutions (*e.g.*, AgNO_3_ and HAuCl_4_) under controlled conditions (temperatures ranging from 25 to 80 °C, pH 7–11, and reaction times from 1 h to overnight). Within these extracts, various phytochemicals contribute to both reduction and stabilization of the prepared nanoparticles. For instance, phenolics and flavonoids are well-known for their reducing capacity and also function as excellent capping agents. Proteins and tannins also play significant roles in stabilizing the nanoparticles. The identification of these reducing and stabilizing agents was predominantly inferred from FTIR analyses.

### 3.1. Metallic nanoparticles synthesized using *Pistacia atlantica* extracts

Sadeghi *et al.* (2015) reported the green synthesis of AgNPs employing an aqueous extract attained from the leaves of *P. atlantica* and investigated their antimicrobial properties.^60^ The extract was prepared from 2 g of dried leaf powder in 25 mL of water with 2 mL methanol, shaken for 1 h, and filtered. For nanoparticle formation, 1 mL of the extract was added to 10 mL of 1 mM AgNO_3_ solution and shaken at room temperature. The reaction was completed within 35 min, as evidenced by a color shift from yellow to deep red. AgNPs were then purified by centrifugation at 10 000 rpm for 15 min, washed, and dried at 60 °C for 24 h.

The formation of the AgNPs could further be confirmed by several characterization techniques, including energy dispersive X-ray analysis (EDX), SEM, TEM, XRD, UV-visible spectroscopy, and FTIR. The size of the obtained green-synthesized AgNPs was in a range of 10–50 nm with a monodisperse appearance as determined by TEM. SEM analysis could reflect a range size of 25–40 nm with few aggregations observed. Additionally, XRD could confirm the crystalline and the cubic structure of the metallic green-synthesized AgNPs with a size of 27 nm. The AgNPs exhibited a remarkable stability profile, showing a substantial increase in AgNPs' zeta potential from −21.7 mV to −64 mV over a wide range of pH (7 to 11), with the highest stability observed at pH 11 (−64.3 mV).^60^ More importantly, the antimicrobial activity of the green-synthesized AgNPs was examined against *S. aureus* using SEM, in which the bacteria treated with AgNPs showed significant damage compared to bacteria treated with the free extract.^60^ These findings refer to the effective potential of the AgNPs as antibacterial agents prepared from an eco-friendly and safe source of a natural extract (*P. atlantica*) compared to other conventional methods of preparation. In contrast, while the study demonstrated the green synthesis and antibacterial potential of *P. atlantica*-derived AgNPs, further investigation into their long-term stability and potential cytotoxicity to human cells is needed to support their clinical translation.

Molaei *et al.* (2018) described the synthesis of palladium nanoparticles (PdNPs) using *P. atlantica* fruit broth *via* a bioreduction route.^61^ PdCl_2_ was first solubilized in 0.1 M HCl, diluted to 1 mM, and then 100 mL of this solution was reacted with 15 mL of freshly prepared extract. The reaction was conducted in a water bath at temperatures ranging from 25 to 85 °C in the dark, where 85 °C was identified as the optimal temperature based on nanoparticle yield, dispersion, and UV-Vis absorbance. Following their synthesis, PdNPs were purified *via* centrifugation at 10 000 rpm for 20 min. Phenolic and triterpenoid compounds in the extract were suggested as reducing agents, while sugars such as starch acted as stabilizers. A proposed mechanism involved reduction of Pd(ii) to Pd(0) through oxidation of hydroxyl groups (polyols), with a supporting pH drop from 3.0 to 1.6 during the reaction.^61^

The characteristics of PdNPs were investigated using SEM, TEM, UV-Vis, XRD, FTIR, and EDX spectroscopy.^61^ PdNPs showed spherical shapes, crystalline structures, sizes of <15 nm, and a surface charge of −24.5 mV. Interestingly, the results proposed that the oxidation of hydroxyl groups within the *P. atlantica* biomass facilitated the reduction of Pd(ii) ions and hence the formation of PdNPs. Furthermore, stabilizing agents, such as starch and fatty acids present in the *P. atlantica* extract, might have hindered the aggregation of the fabricated nanoparticles.^61^ While the study offered a bio-inspired approach for synthesizing PdNPs using a *P. atlantica* aqueous extract, future research to investigate their biocompatibility and interactions with biomolecules for targeted bioapplications is required.

The remarkable antioxidant activity of the extracts derived from *P. atlantica* (PS) and *Punica granatum* (PO) owing to their rich content of phenolic compounds and flavonoids inspired a study (2021) to investigate the therapeutic potential of AgNPs synthesized utilizing *P. atlantica* leaf extract (PS–AgNPs) and *P. granatum* peel extract (PO–AgNPs) against acute liver failure induced by thioacetamide (TAA) in rats.^62^ The AgNPs were synthesized using aqueous extracts of *Pistacia atlantica* and *Punica granatum* as reducing and capping agents. A 1 mM AgNO_3_ solution was prepared by dissolving 0.017 g AgNO_3_ in 100 mL distilled water. Then, 1 mL of plant extract was added to 50 mL of this solution and incubated at room temperature under static conditions in the dark to prevent light-induced activation. The reduction of Ag^+^ to AgNPs was visually confirmed by a color change from colorless to brown. The nanoparticles were purified *via* centrifugation and dried using a vacuum dryer. UV-Vis analysis confirmed nanoparticle formation and TEM showed spherical particles ranging in size from 7 to 17 nm.^62^

**Fig. 2:**
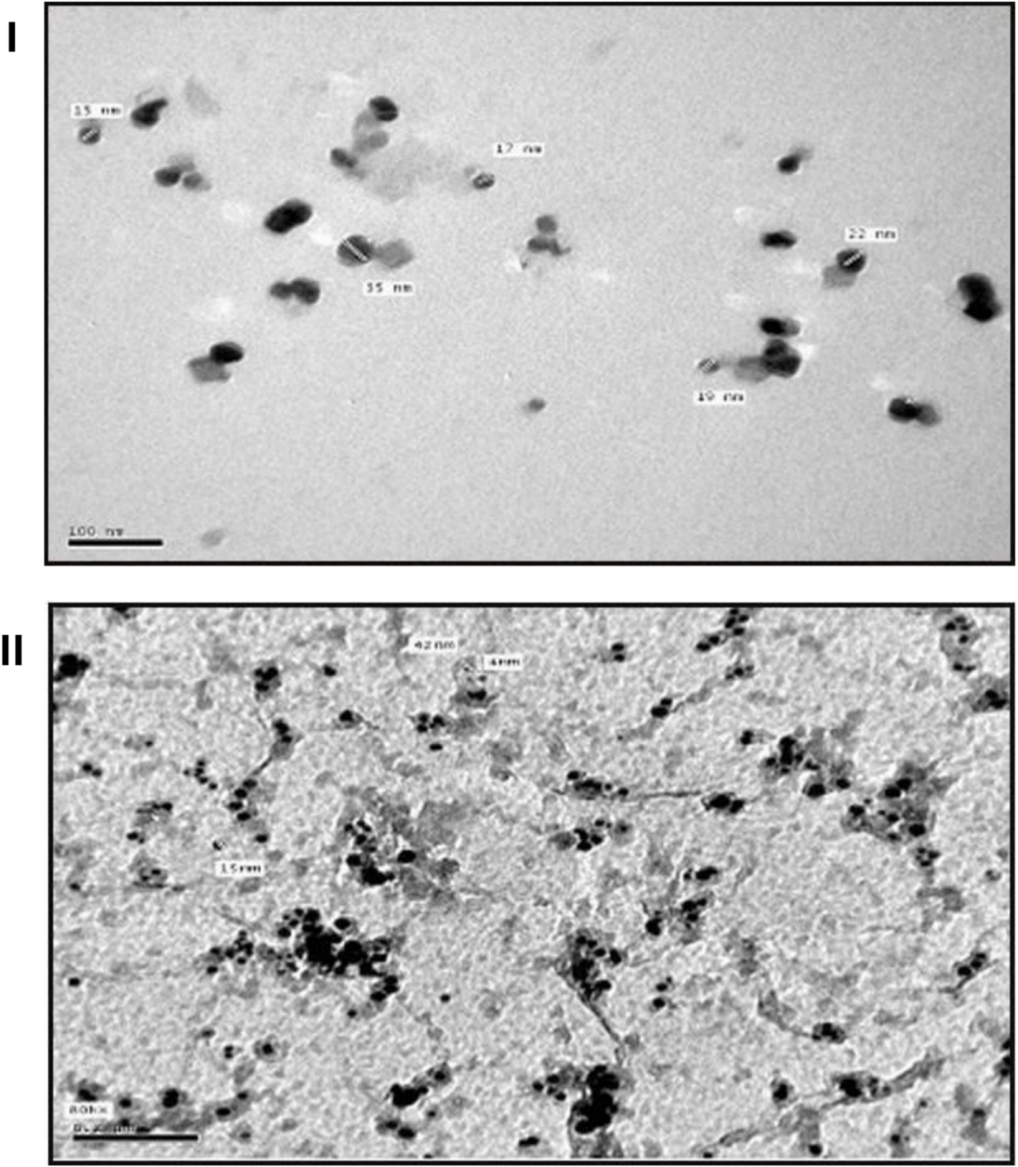
(I) TEM image of AgNPs synthesized exploiting the extract of *P. granatum* peels, showing spherical nanoparticles with a mean diameter of 17 nm; (II) TEM image of AgNPs synthesized employing the extract of *P. atlantica* leaves, showing spherical nanoparticles with a mean diameter of 15 nm.^62^ Reprinted from ref. 62. Copyright Egyptian Journal of Chemistry 2021.

**Fig. 3:**
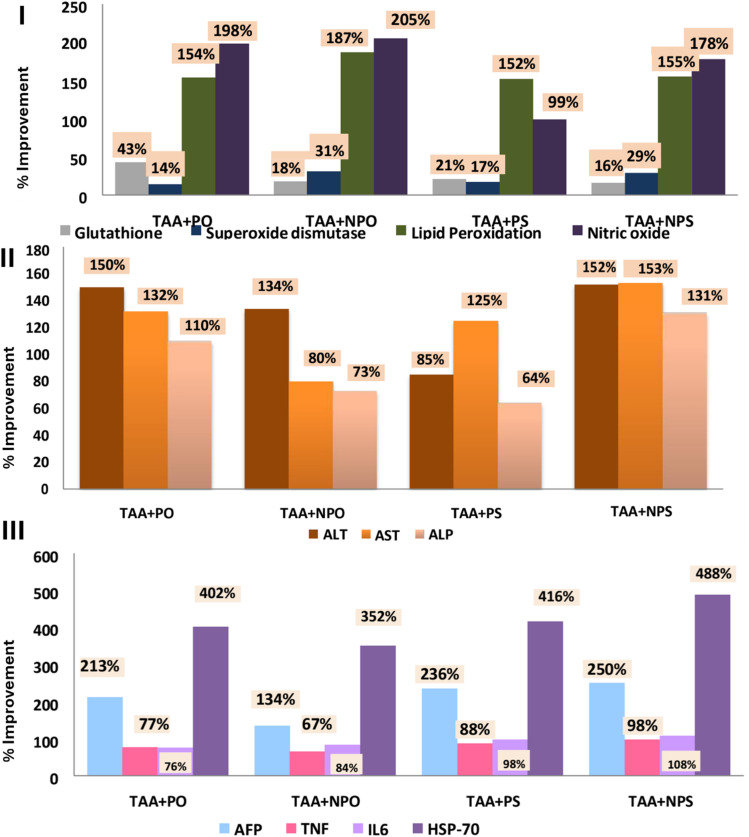
(I) Therapeutic effects, expressed as % improvement in oxidative stress markers (glutathione, superoxide dismutase, lipid peroxidation, and nitric oxide), of the *P. granatum* peel extract (PO), the *P. atlantica* leaf extract (PS), and their corresponding AgNPs (NPO and NPS, respectively) against thioacetamide (TAA)-induced acute liver toxicity in rats. (II) Therapeutic effects, expressed as % improvement in liver serum markers (ALT, ALP, and AST) of PO, PS, NPO, and NPS against TAA-induced acute liver toxicity in rats. (III) Therapeutic effects, expressed as % improvement in inflammatory cytokines (AFP, TNF, IL6, and HSP-70) of PO, PS, NPO, and NPS against TAA-induced acute liver toxicity in rats. Reprinted from ref. 62. Copyright Egyptian Journal of Chemistry 2021.

Furthermore, the formation of PS–AgNPs and PO–AgNPs was confirmed using TEM and SEM analyses, where the nanoparticles showed a spherical shape, sizes of 7–17 nm, and zeta potentials from −10.6 to −23.6 mV, reflecting high stability (Fig. 2).^62^ Treatments with PO–AgNPs and PS–AgNPs significantly improved liver function compared to the TAA group, as demonstrated by reductions in serum levels of ALT activity (by 54.04% and 62.28%, respectively), AST activity (by 27.61% and 52.93%, respectively), and ALP activity (by 34.04% and 60.91%, respectively). On the other hand, treatments with PO and PS extracts enhanced the liver function, compared to the TAA group, as shown by reductions in serum levels of ALT activity (by 60.46% and 34.43%, respectively), AST activity (by 45.77% and 43.38%, respectively), and ALP activity (by 46.81% and 29.68%, respectively) (Fig. 3II). Furthermore, PO–AgNP and PS–AgNP treatments significantly increased the levels of glutathione by 28.36% and 27.26%, respectively, compared to the TAA group. On the other hand, treatments with PO and PS extracts could also substantially increase the levels of glutathione by 93.58% and 31.83%, respectively, compared to the TAA group (Fig. 3I). Additionally, PO–AgNP and PS–AgNP treatments decreased the levels of the pro-inflammatory marker tumor necrosis factor-alpha (TNF-α) by 25.14% and 36.87%, respectively, whereas treatments with PO and PS reduced the levels of TNF-α by 29.62% and 33.2%, respectively, compared to the TAA group. Also, interleukin-6 (IL-6) was reduced by 33.12% (PO–AgNPs) and 42.78% (PS–AgNPs), while treatments with PO and PS reduced the levels of IL-6 by 29.91% and 38.48%, respectively, compared to the TAA group (Fig. 3III). Histopathological analysis confirmed reduced necrotic areas and promoted liver tissue repair in PO–AgNP, PS–AgNP, PO, and PS treated groups compared to the TAA group.^62^ These findings suggest that PO–AgNPs and PS–AgNPs possess promising therapeutic potential for treating acute liver failure due to their ability to improve liver function and reduce oxidative stress and inflammation, highlighting their potential and warranting further investigation into their therapeutic applications and mechanisms.

**Fig. 4:**
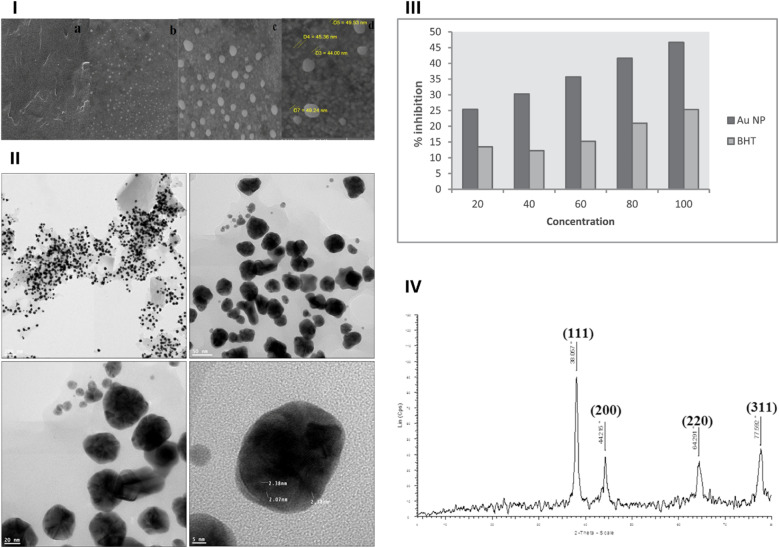
(I) SEM images of the *P. atlantica* extract (a) and green-synthesized AuNPs (b–d), at various magnifications; (II) TEM images of green-synthesized AuNPs at various magnifications; (III) antioxidant activity of green-synthesized AuNPs; (IV) XRD pattern of green-synthesized AuNPs.^63^ Reprinted with permission from ref. 63. Copyright Elsevier 2018.

Hamelian *et al.* (2019) described a green synthesis approach for AuNPs exploiting an aqueous extract of *P. atlantica* (fruits and leaves) to investigate their antimicrobial, antioxidant, and cytotoxic activities.^63^ HAuCl_4_ was initially treated with the *P. atlantica* extract at room temperature, leading to the fabrication of AuNPs. The reduction of Au^3+^ ions is attributed to the bioactive compounds present in the *P. atlantica* extract, primarily phenolics and flavonoids.^63^ Briefly, the nanoparticles were synthesized using aqueous extracts of *P. atlantica* leaves, fruits, and their mixture as reducing and stabilizing agents. Each extract (10 mL) was mixed with 100 mL of 1 mM HAuCl_4_ solution and stirred at room temperature. The reaction initiated rapidly, with a color change observed within 10–60 s depending on the extract type (fruit < mixture < leaves). The mixtures were stirred for 1 h, centrifuged at 12 000 rpm for 15 min, washed multiple times, and oven-dried at 50 °C. UV-Vis analysis showed that the extract type influenced the particle size: the leaf extract yielded ∼ 30 nm AuNPs (*λ*_max_ = 520 nm, red), the mixture yielded ∼ 40 nm (*λ*_max_ = 530 nm, light purple), and the fruit extract yielded ∼ 50 nm particles (*λ*_max_ = 540 nm, dark purple), suggesting that phytochemical composition affected nanoparticle characteristics.^63^ TEM revealed spherical AuNPs with a mean size of 2.07–2.38 nm, whereas SEM analysis revealed AuNPs with a mean of 40–50 nm owing to agglomeration induced during sample preparation (Fig. 4I and II). The crystalline structures of AuNPs were confirmed using XRD (Fig. 4IV).^63^ The AuNPs exhibited significant antimicrobial effects against *B. subtilis*, *S. aureus*, *P. aeruginosa*, and *E. coli* bacteria as shown by the agar diffusion test. Interestingly, MIC values were found to be 31.25 μg mL^−1^ for *B. subtilis*, 7.81 μg mL^−1^ for *S. aureus*, 3.9 μg mL^−1^ for *P. aeruginosa*, and 7.81 μg mL^−1^ for *E. coli*. Additionally, minimum bacterial concentration (MBC) values were reported with 15.62 μg mL^−1^ for *S. aureus*, 62.5 μg mL^−1^ for *B. subtilis*, 62.5 μg mL^−1^ for *P. aeruginosa*, and 15.62 μg mL^−1^ for *E. coli*. Moreover, all four bacteria tested were inhibited by the synthesized AuNPs employing agar disk diffusion assay. The strongest inhibition zones, around 20–23 mm in diameter, were observed against all bacteria exploiting a 31 μg mL^−1^ concentration of AuNPs. Even at a much lower concentration (3 μg mL^−1^), all bacteria showed some inhibition, with an inhibition zone around 8 mm in diameter. The antioxidant activity of the synthesized AuNPs was determined using DPPH free radical scavenging assay, demonstrating a dose-dependent scavenging activity, reaching ∼45% employing a concentration of 80 μg mL^−1^ as compared to butylated hydroxytoluene that showed around 20% scavenging activity at the same concentration. This suggests the potential role of AuNPs biosynthesized using the *P. atlantica* extract for mitigating oxidative stress (Fig. 4III). Furthermore, the cytotoxicity of the synthesized AuNPs was evaluated against HeLa cells using an MTT assay. The treated cells, even at the highest tested concentration of 200 μg mL^−1^, depicted no cytotoxic effect after 48 h.^63^ Further testing on normal cells is necessary to assess the safety profile of the AuNPs. Overall, the synthesis of AuNPs using the *P. atlantica* extract remarkably enhanced their therapeutic potential as broad-spectrum antibacterial agents and antioxidants. However, further investigation is required to elucidate their mechanism of action and ensure their biocompatibility for *in vivo* applications.

**Fig. 5:**
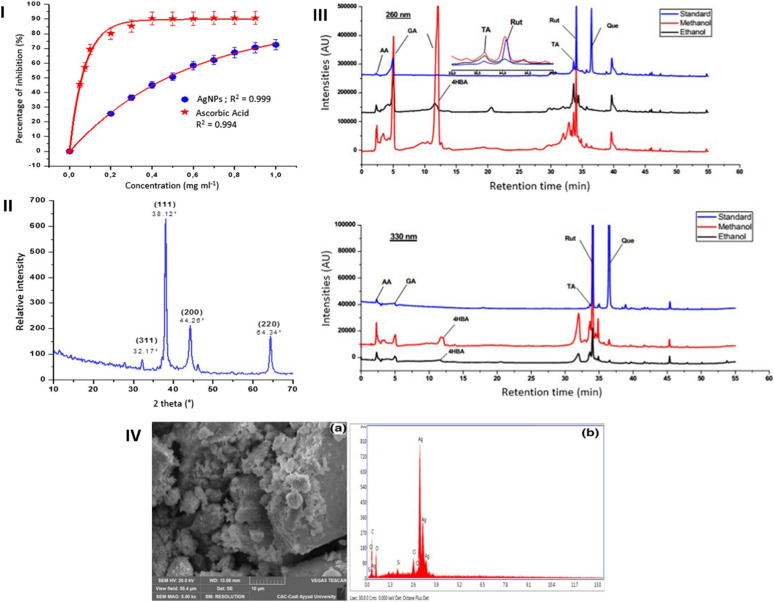
(I) Antioxidant activity of green-synthesized AgNPs compared to a standard of ascorbic acid; (II) XRD spectrum pattern of green-synthesized AgNPs; (III) HPLC chromatograms of the *P. atlantica* methanolic extract compared to several standards of quercetin (Que), rutin (Rut), tannic acid (TA), gallic acid (GA), 4-hydroxybenzoic acid (4-HBA), and ascorbic acid (AA); (IV) SEM image (a) and EDX spectrum (b) of green-synthesized AgNPs.^64^ Reprinted with permission from ref. 64. Copyright Springer Nature 2021.

The study by Khiya *et al.* (2021) investigated the synthesis of AgNPs employing a methanolic extract of *P. atlantica* leaves and evaluated their antibacterial and antioxidant activities.^64^ Briefly, an aqueous extract of *P. atlantica* was prepared by boiling 5 g of plant powder with 100 mL of distilled water at 60 °C for 30 min, followed by filtration. The resulting extract was stored and later used as both a reducing and stabilizing agent for AgNPs synthesis. AgNPs were synthesized using 2.5 mL of *P. atlantica* methanolic leaf extract added to 1 mM AgNO_3_ solution, followed by incubation at 60 °C for 1 h in the dark. The mixture was then cooled to 25 °C for 24 h, centrifuged at 3600 rpm for 30 min, washed, and dried at 90 °C for 1 h. A visible color change from yellow to brown confirmed nanoparticles formation. UV-Vis analysis showed a broad surface plasmon resonance (SPR) peak at 468 nm, characteristic of AgNPs. HPLC-PDA analysis identified several phenolics in the extract, with gallic acid, 4-hydroxybenzoic acid, and rutin being dominant, while quercetin appeared in lower concentrations. These phenolic compounds were implicated as both reducing and capping agents in the nanoparticles synthesis.^64^ The crystallinity of the AgNPs was confirmed using X-ray crystal analysis (Fig. 5II). The presence of the elemental signature of Ag was further confirmed by SEM-EDX (Fig. 5IV). FTIR spectroscopy of the AgNPs revealed characteristic absorption bands, which differed from those of the raw plant extract. These observed shifts and changes in band positions indicated the formation of AgNPs and, particularly, the involvement of various functional groups from the plant phytochemicals, such as hydroxyl (O–H) and carbonyl (C=O) groups, in the bioreduction and stabilization process.

The green synthesized AgNPs depicted significant antioxidant effects, using DPPH assay, showing an IC_50_ of 0.47 mg mL^−1^ (Fig. 5I). Furthermore, the prepared AgNPs effectively controlled the growth of *Acinetobacter baumannii*, *S. aureus*, and *E. coli*, by employing a disk diffusion test, and resulted in inhibition zones of 11 mm, 12 mm, and 13 mm, respectively. HPLC analysis revealed the presence of various antioxidant compounds in the ethanolic and methanolic extracts of *P. atlantica*, including quercetin, rutin, tannic acid, gallic acid, and ascorbic acid (Fig. 5III).^64^ Further research including calculation of MIC values for different bacterial species and investigation of the related inhibitory mechanisms is required to further support the utilization of the synthesized AgNPs as antibacterial and antioxidant agents.

Golabiazar *et al.* (2019) explored an eco-friendly and green synthesis method to fabricate AgNPs exploiting the leaf extract of *P. atlantica*, as a stabilizer, capping, and reducing agent, and investigated the potential of the obtained AgNPs as antibacterial agents.^65^ The AgNPs were synthesized using a *P. atlantica* leaf extract prepared by boiling 100 g of dried leaf powder in 500 mL of distilled water for 30 min. Then, 50 mL of this extract was added dropwise to 50 mL of 0.003 mL per L AgNO_3_ solution under constant stirring at 80 °C. A visible color change from white to yellowish brown occurred during heating (peak at 445–450 nm), indicating the formation of AgNPs through excitation of SPR.^65^ XRD confirmed the crystalline structures of the obtained nanoparticles, while showing a mean size of 17 to 18 nm. TEM analysis exhibited spherical shaped AgNPs with a size of <50 nm and EDX analysis showed aggregates that represent the AgNPs. Similarly, FTIR analysis revealed notable differences in the location and shape of some signals, indicating interaction occurred between AgNO_3_ and the included sites of biomolecules responsible for developing AgNPs. Also, SEM images supported the formation of aggregates of AgNPs with spherical shapes.^65^

**Fig. 6:**
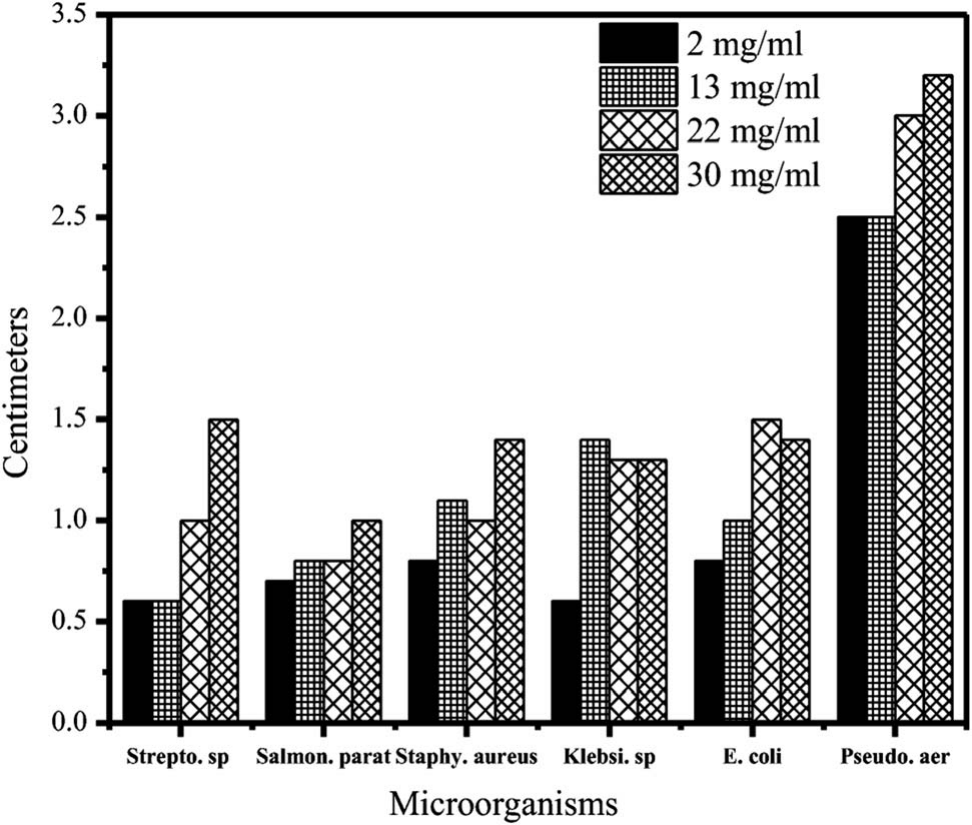
Effects of green-synthesized AgNPs' antimicrobial activities, employing various concentrations, on the inhibition zone of the specified bacterial strains.^65^ Reprinted with permission from ref. 65. Copyright Springer Nature 2019.

The antimicrobial effects of AgNPs were investigated using the disk diffusion method, showing promising activity even at the lowest concentration of the AgNPs (2 mg mL^−1^) utilized against *Salmonella paratyphi B*, *Streptococcus pyogenes*, *P. aeruginosa*, *Klebsiella pneumoniae*, *S. aureus*, and *E. coli* (Fig. 6). The zone of inhibition ranged from 6 mm (*S. pyogenes* and *K. pneumoniae*) to 25 mm (*P. aeruginosa*), with diameters of 7 mm (*S. paratyphi B*) and 8 mm (*S. aureus* and *E. coli*) observed for other strains.^65^ While these results highlight the antibacterial potential of such green-synthesized AgNPs, future studies should explore the MIC for each bacterial strain. Additionally, comparisons with standard antibiotics could provide valuable context regarding the relative efficacy of these AgNPs.

**Fig. 7:**
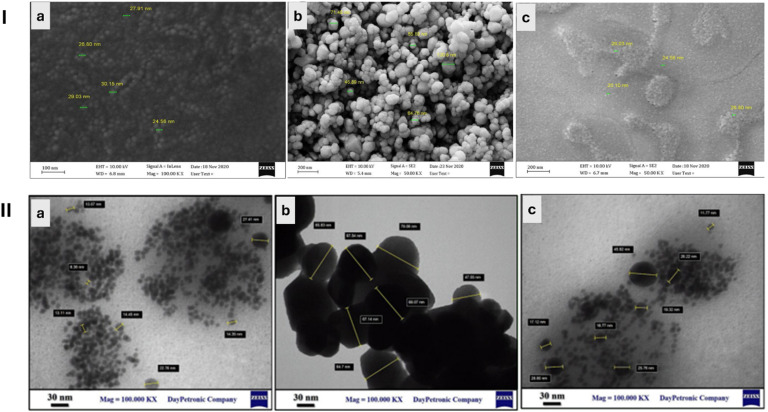
SEM (I) and TEM (II) images of AgNPs (a), ZnONPs (b), and AgZnONPs (c) synthesized using the resin extract of *P. atlantica*.^66^ Reprinted with permission from ref. 66. Copyright Elsevier 2021.

**Fig. 8:**
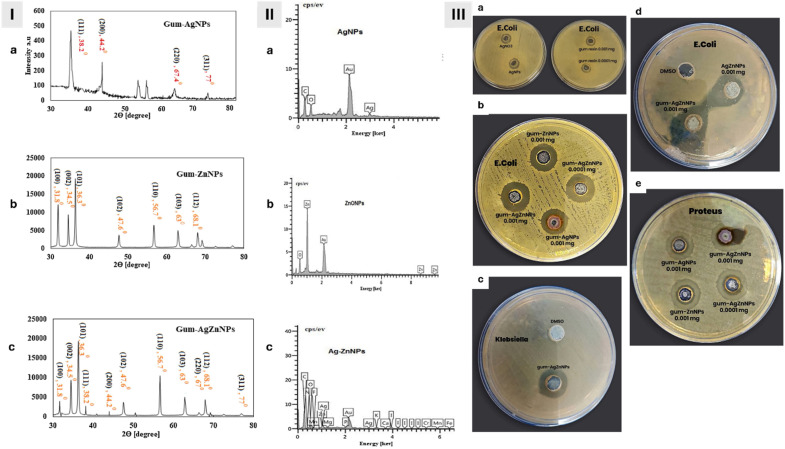
XRD (I) and EDX (II) spectra of AgNPs (a), ZnONPs (b), and AgZnONPs (c) synthesized using the resin extract of *P. atlantica*; (III) antimicrobial activities, expressed as zones of inhibition, of AgNPs, ZnONPs, and AgZnONPs against *E. coli* (a, b and d), *K. pneumoniae* (c), and *P. mirabilis* (e).^66^ Reprinted with permission from ref. 66. Copyright Elsevier 2021.

Jomehzadeh *et al.* (2021) described a simple method for synthesizing AgNPs, zinc oxide nanoparticles (ZnONPs), and silver-zinc oxide nanocomposites (AgZnONPs) utilizing the resin extract of *P. atlantica* as both a reducing and a capping agent for the nanoparticles.^66^ AgNPs, ZnONPs, and AgZnONPs were synthesized using *P. atlantica* resin extract, which served as both reducing and stabilizing agents. The resin was dissolved in 10% ethanol and DMSO and then used at various concentrations. For AgNPs, 20 mL of resin extract was mixed with 1 mL of 0.001 M AgNO_3_ and incubated at 70 °C for 5 min. ZnONPs were prepared using 3 mL resin extract + 47 mL of 0.05 mM Zn(NO_3_)_2_ at 70 °C for 150 min, while AgZnONPs were synthesized using 5 mL resin extract with 10 mL Zn(NO_3_)_2_ (0.05 M) + 5 mL AgNO_3_ (0.01 M), stirred at 70 °C for 60 min. Nanoparticles were purified by centrifugation and dried at 80–150 °C. Phytochemical analysis confirmed the presence of high phenol (149.35 mg), ascorbic acid (20.79 mg), and moderate flavonoid (56.16 mg) and reducing sugar (21.11 mg) content, supporting their role as reducers and stabilizers.^66^ Moreover, the formation and characteristics of the developed nanoparticles were confirmed using UV-Vis, TEM, FTIR, XRD, SEM, and EDX (Fig. 7 and 8). UV-Vis confirmed the color changes and the wavelengths of the synthesized nanoparticles corresponding to the reduction of Zn^2+^ and Ag^+^ to metals (Zn and Ag) were 430 nm for AgNPs and 320–360 nm for ZnONPs and AgZnONPs. TEM analysis showed a mean size of <50 nm for AgNPs and AgZnONPs, accompanied by spherical shapes, and a mean size of <70 nm for ZnONPs, with polyhedral shapes (Fig. 7). XRD and FTIR analyses confirmed the crystalline and chemical structures of the developed nanoparticles (Fig. 8).^66^

More importantly, the antimicrobial activities of all nanoparticles synthesized using *P. atlantica* extract were assessed against four bacterial strains: *K. pneumoniae*, *E. coli*, *Proteus mirabilis* (*P. mirabilis*), and *S. aureus* (Fig. 8). Interestingly, all nanoparticles exhibited some level of inhibition at a concentration of 0.001 mg mL^−1^. For the *P. atlantica* resin extract alone, the extract showed inhibition zones ranging from 7 mm (*K. pneumoniae*) to 11 mm (*P. mirabilis*). On the other hand, AgNPs prepared using the extract displayed slightly larger inhibition zones (12.5–15 mm) compared to AgNPs alone (10–15 mm) across all bacteria, suggesting a potential improvement due to the plant extract. Additionally, ZnONPs prepared using the extract exhibited greater inhibition zones (13.6–17.5 mm) compared to ZnONPs alone (11.3–13.5 mm), referring further to a potential influence of the extract. However, the nanocomposite of AgZnONPs prepared using the extract exerted the strongest antibacterial activity compared to all other nanoparticles. AgZnONPs prepared using the extract showed inhibition zones of 20 ± 1.2 mm (*E. coli*), 15 ± 0.08 mm (*K. pneumoniae*), 21 ± 0.002 mm (*P. mirabilis*), and 16 ± 0.3 mm (*S. aureus*) compared to AgZnONPs alone, which exhibited zones of inhibition of 15.2 ± 0.007 mm (*E. coli*), 12 ± 0.05 mm (*K. pneumoniae*), 13 ± 0.004 mm (*P. mirabilis*), and 12 ± 0.06 mm (*S. aureus*).^66^ These findings indicate a potential synergistic effect between Ag and ZnO in the nanocomposite prepared, leading to enhanced antibacterial activity. Overall, the study highlighted the promising potential of these green-synthesized nanoparticles, particularly AgZnONPs derived from the *P. atlantica* extract, due to their broad-spectrum antimicrobial properties against these pathogens, and further studies exploring the mechanism of action and *in vivo* efficacy are encouraged for a more comprehensive evaluation.

Veisi *et al.* (2019) investigated a green approach for reduced graphene oxide–AgNPs (RGO–AgNPs) nanocomposite synthesis mediated by the leaf extract of *P. atlantica*, as a reducing and stabilizing agent.^67^ For the synthesis, 10 g of dried leaf powder was boiled in 300 mL of deionized water for 30 min and filtered. This extract was added to 100 mg of GO in 100 mL water, sonicated for 30 min, and refluxed for 5 h to reduce GO and anchor the extract. After washing and redispersion, 20 mg of AgNO_3_ in 20 mL water was added to the mixture, which was refluxed and stirred at 100 °C for 2 h. The silver content in the final nanocomposite was 0.23 mmol g^−1^. Phytochemical analysis confirmed the presence of flavonoids, phenolic glycosides, tannins, coumarins, and saponins in the extract, which likely mediated the reduction of both GO and Ag^+^*via* π-electron interactions and chelation mechanisms.^67^ This nanocomposite showed a peak at 235–270 nm, which corresponds to RGO, and another at 423 nm indicative of AgNPs. XRD, EDX, and FTIR confirmed the crystalline and chemical structures of the nanoparticles. The FTIR analysis indicated the absence of the carbonyl (C=O) peak associated with the carboxyl group, suggesting effective reduction of silver ions. However, several functional groups were observed on the RGO surface, including hydroxyl (OH), oxygen-containing groups, and aliphatic/aromatic C–H, derived from the flavonoids, tannins, and phenolic compounds in the extract. These compounds, likely adsorbed through π-electron interactions, played a crucial role in the reduction process and subsequent stabilization of silver ions on the RGO surface. The XRD analysis showed 2*θ* of 10–20, corresponding to RGO, and 2*θ* values of 38.2, 44.5, 64.6 and 77.4°, corresponding to AgNPs. SEM and TEM analyses exhibited a uniform distribution of small AgNPs (with a size range of 12.75–73.35 nm) on the surfaces of RGO, in which the RGO was further covered by a thin layer of biomolecules derived from the extract, potentially attributed to the AgNPs reduction and stabilization.^67^

Moreover, RGO–AgNPs exhibited a concentration-dependent antibacterial activity against a broad spectrum of bacteria. At the highest concentration tested (64 mg mL^−1^), the nanocomposite displayed significantly larger inhibition zones against all tested bacteria compared to the *P. atlantica* extract alone. For instance, RGO–AgNPs produced a zone of inhibition of 64.2 ± 1.3 mm against *Staphylococcus saprophyticus*, while the extract showed no inhibition.^67^ These results were further confirmed by MIC and MBC values. RGO–AgNPs suppressed the growth of various bacteria even at concentrations reaching 1 mg mL^−1^. Particularly, RGO–AgNPs suppressed *S. aureus* growth with an MIC of 2 mg mL^−1^ and completely eliminated its growth with an MBC of 4 mg mL^−1^ (MBC, the minimum bactericidal concentration, is the lowest concentration of an antimicrobial agent required to kill a specific microorganism). Similarly, the nanocomposite inhibited *S. saprophyticus*, *Streptococcus pyogenes*, and *B. subtilis* at a low MIC of 1 mg mL^−1^, whereas the MBC values were 1 mg mL^−1^, 2 mg mL^−1^, and 1 mg mL^−1^, respectively. These findings indicate strong antimicrobial properties against these Gram-positive strains.^67^ For Gram negative bacteria, the MIC values were slightly higher, ranging from 1 mg mL^−1^ for *P. aeruginosa* to 2 mg mL^−1^ for *E. coli* and 4 mg mL^−1^ for *Salmonella typhimurium* and 8 mg mL^−1^ for *Proteus mirabilis*. The MBC values against the same strains followed a similar pattern, with 4 mg mL^−1^, 1 mg mL^−1^, 4 mg mL^−1^, and 8 mg mL^−1^, respectively. These findings depict a broad-spectrum efficacy of the RGO–AgNPs nanocomposite synthesized utilizing the leaf extract of *P. atlantica* against various bacterial strains.^67^ The incorporation of AgNPs significantly enhanced the potency against a broad spectrum of bacteria, showing the promise of the developed nanocomposite for future antibacterial applications. Future *in vivo* studies while exploring the underlying mechanisms of action and safety of the nanocomposite are warranted.

Hamelian *et al.* (2020) fabricated AgNPs using an aqueous *P. atlantica* leaf extract as a reducing and stabilizing agent.^68^ The leaf extract was prepared by soaking 100 g of powdered leaves in 1000 mL distilled water for 24 h, followed by concentration at 40 °C using a rotary evaporator. For AgNPs synthesis, 2 g of the extract was dissolved in 20 mL of deionized water, and 2.5 mL of the solution was added to AgNO_3_ (5 × 10^−4^ M). The reaction mixture was heated at 80 °C under stirring for 24 h. The visual color change from greenish yellow to black after 48 h confirmed nanoparticles formation. UV-Vis spectroscopy showed a strong SPR peak at 440 nm, attributed to the hydroxyl moieties in the extract, corresponding to the reduction of Ag^+^ to Ag, leading to successful AgNPs formation. The synthesis was pH-responsive and affected by volumetric ratios (0.05–0.6) and time.^68^ Furthermore, AgNPs synthesis was also confirmed by FTIR, TEM, XRD, EDX, SEM, AFM, and TGA. Both TEM and SEM analyses depicted AgNPs with spherical shapes coupled with a mean size of 40–50 nm. TGA analysis depicted a well-stable thermal profile of the nanoparticles with a total weight loss of 38.85% over a temperature range of 20–800 °C.^68^

**Fig. 9:**
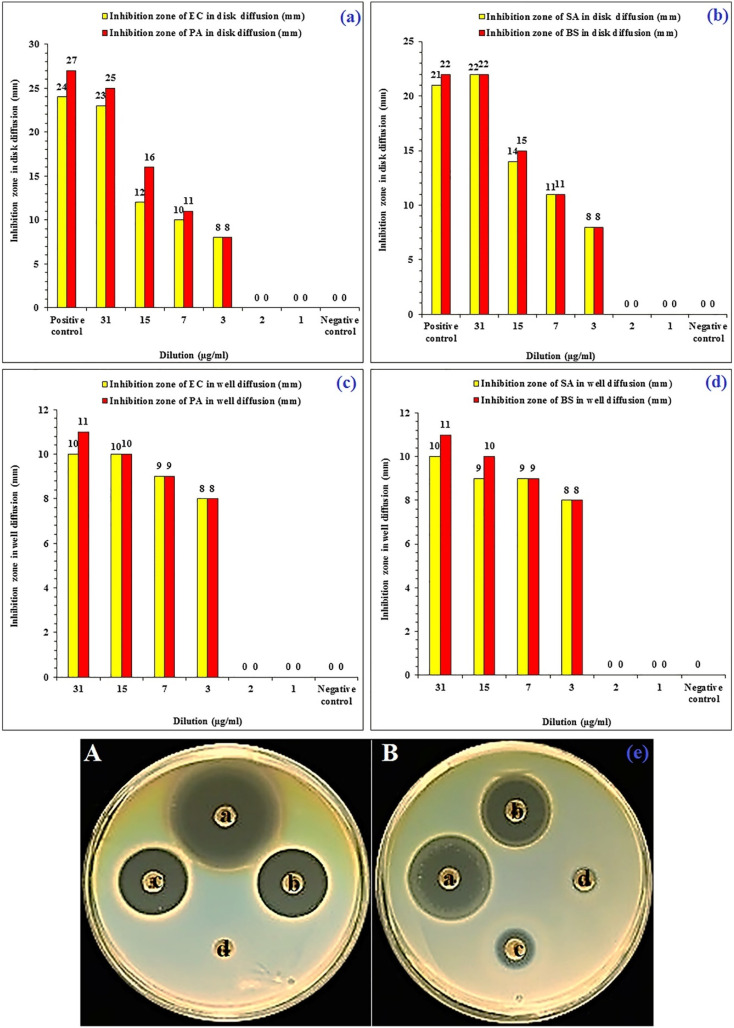
Antibacterial effects of the synthesized AgNPs. (a and c) Growth inhibition zones against *E. coli* (agar diffusion test) and *P. aeruginosa* (agar disk diffusion) treated with various concentrations of green-synthesized AgNPs. (b and d) Diameters of the growth inhibition zones against *S. aureus* (agar disk diffusion test) and *B. subtilis* (agar disk diffusion) treated with various concentrations of green-synthesized AgNPs. (e) Comparison of the diameters of the growth inhibition zones against *P. aeruginosa* (A) and *S. aureus* (B) treated with different concentrations of green-synthesized AgNPs, employing an agar disk diffusion test.^68^ Reprinted with permission from ref. 68. Copyright John Wiley and Sons 2019.

More importantly, the radical scavenging activity of the AgNPs was assessed employing a DPPH antioxidant assay, showing promising scavenging property of free radicals (87.71%) at a concentration of 100 μg mL^−1^ of AgNPs, compared to a reference of butylated hydroxytoluene, which exhibited only 25.32% scavenging activity at the same concentration.^68^ Additionally, the safety profile of AgNPs was investigated against normal endothelial cells (HUVECs), exhibiting remarkable viability of the treated cells (>80% viable cells) even at the highest concentration tested (200 μg mL^−1^).^68^ Finally, the AgNPs demonstrated significant antimicrobial effects against *E. coli*, *B. subtilis*, *P. aeruginosa*, and *S. aureus*. No inhibition zones were noticed at 1 and 2 μg mL^−1^, but large inhibition zones were depicted at 31 μg mL^−1^, employing the agar well and disk diffusion assays (Fig. 9). *P. aeruginosa* showed the highest susceptibility and lowest MIC (1 μg mL^−1^) and MBC (3 μg mL^−1^) values. *E. coli* required slightly higher concentrations for growth inhibition (MIC of 3 μg mL^−1^) and eradication (MBC of 3 μg mL^−1^) compared to *P. aeruginosa*. Nevertheless, both *B. subtilis* and *S. aureus* showed similar MIC values (7 μg mL^−1^) but differed in their MBCs, with *S. aureus* requiring a higher concentration (15 μg mL^−1^) for complete eradication compared to *B. subtilis* (7 μg mL^−1^).^68^ These results demonstrate the potential of AgNPs synthesis using a *P. atlantica* leaf extract, highlighting their promising antioxidant activity, safe profile against normal cells, and antibacterial activity against common pathogens, showing promise for biomedical applications.

### 3.2. Metallic nanoparticles synthesized using *Pistacia vera* L. extracts

**Fig. 10:**
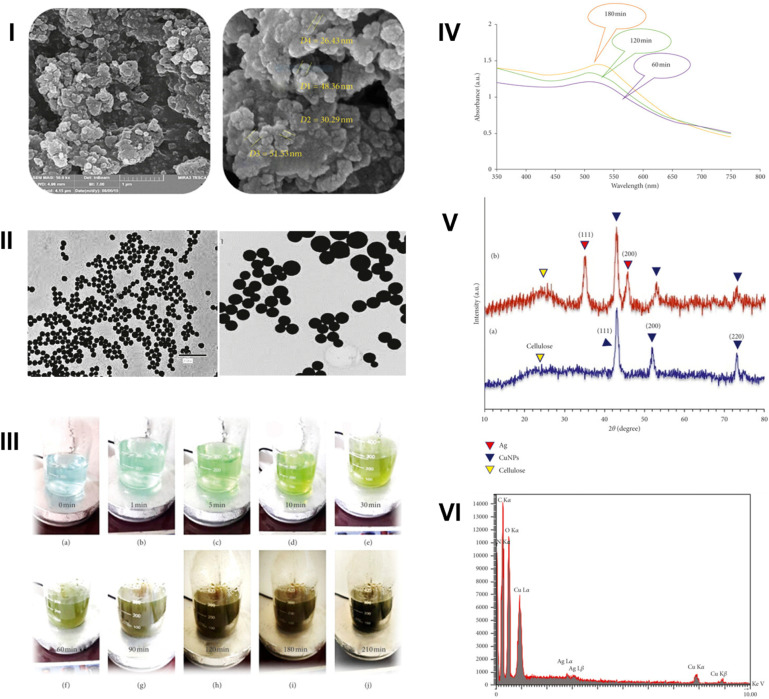
SEM (I) and TEM (II) images of CuNPs green-synthesized using the hull extract of *P. vera*. (III) Color changes observed at different time intervals throughout the synthesis of CuNPs using the *P. vera* hull extract. (IV) UV-Vis absorption spectrum at different time intervals of CuNPs synthesis. (V) XRD spectra of green-synthesized CuNPs utilizing the *P. vera* extract (a) and CSS NC (b). (VI) EDX profile of the CuNPs/silver Schiff base nanocomposite (CSS NC).^69^ Reprinted from ref. 69. Copyright John Wiley & Sons 2021.

Bakhshi *et al.* (2021) utilized a green synthesis approach for fabricating copper nanoparticles (CuNPs) using a *Pistacia vera* (pistachio) hull extract.^69^ Briefly, dried pistachio hulls (15 g) were sonicated in 150 mL of deionized water at 45 °C for 20 min and centrifuged at 6000 rpm for 30 min. The extract was filtered and stored at 4 °C. For nanoparticles synthesis, 8 mL of the extract was added dropwise to 100 mL of 8 mM copper(ii) acetate solution at 70 °C under stirring. The reaction proceeded for 5 h, during which the color changed sequentially from sky blue to dark olive, indicating CuNPs formation. The obtained mixture was then centrifuged, washed with water, ethanol, and *n*-hexane, and then dried at room temperature, followed by heating at 65 °C for 1 h.^69^ A copper Schiff base nanocomposite (CSS NC) was subsequently prepared. A silver Schiff base ligand (SL) was synthesized from 2-aminophenol and salicylaldehyde in ethanol. An ethanol solution of AgNO_3_ (0.2 M) was added to an ethanol solution of SL (0.1 M) under sonication to form a silver Schiff base complex (CSS). Finally, the aqueous CSS was incorporated into the CuNPs suspension for 1 h under stirring. The CuNPs were characterized by SEM (26–51 nm), TEM (15–45 nm), FTIR, XRD, EDX, and UV-Vis, showing a peak around 500 nm (Fig. 10).^69^

More importantly, both CuNPs and CSS NC exhibited broad-spectrum antimicrobial activity. *E. coli* was most susceptible to CuNPs (MIC 3.125 μg mL^−1^) and CSS NC (MIC 1.56 μg mL^−1^). *A. niger* was also significantly inhibited by CuNPs (MIC 3.125 μg mL^−1^) and CSS NC (MIC 1.56 μg mL^−1^). Other MICs were *S. aureus* (25 μg mL^−1^ CuNPs, 12.5 μg mL^−1^ CSS NC), *B. cereus* (12.5 μg mL^−1^ CuNPs, 6.25 μg mL^−1^ CSS NC), *P. aeruginosa* (6.25 μg mL^−1^ CuNPs, 3.12 μg mL^−1^ CSS NC), and *C. albicans* (6.25 μg mL^−1^ CuNPs, 3.12 μg mL^−1^ CSS NC). These results highlight the promise of *P. vera* hull extract-derived CuNPs and CSS NC as broad-spectrum antimicrobial agents.^69^

### 3.3. Metallic nanoparticles synthesized using *Pistacia lentiscus* extracts

AgNPs were synthesized using a *Pistacia lentiscus* leaf extract. Fresh leaves (5 g) were boiled in 200 mL of 50% ethanol on a steam bath for 15–20 min until the solution turned light green and then filtered and stored at 4 °C. For nanoparticle formation, 30 mL of this extract were mixed with 30 mL of 0.025 M AgNO_3_ solution and incubated at 50 °C. A visible color change indicated AgNPs formation alongside the appearance of a distinct broad peak at 450 nm.^70^ The XRD spectrum confirmed the crystallinity of the obtained AgNPs with a size of 26 nm, whereas the TEM image exhibited a mean size of 24 nm with a spherical shape of the obtained particles.^70^

The antimicrobial activities of the bio-synthesized AgNPs were investigated against *Aspergillus flavus* (*A. flavus*) and *Aspergillus niger* (*A. niger*) and several bacterial strains including *B. subtilis*, *E. coli*, *P. aeruginosa*, *S. aureus*, *Streptococcus faecalis* (*S. faecalis*), and *Neisseria gonorrhoeae* (*N. gonorrhoeae*). Utilizing the disc diffusion assay, the inhibition zones were found to be 13 mm, 13 mm, 14 mm, 14 mm, 13 mm, and 12 mm for *E. coli*, *S. aureus*, *P. aeruginosa*, *B. subtilis*, *S. faecalis*, and *N. gonorrhoeae,* respectively. While the synthesized AgNPs exhibited remarkable activity against *A. flavus* with an inhibition zone diameter of 11 mm, they failed to reveal any significant activity against *A. niger*.^70^ The improved properties of the developed nanoparticles (small size with a larger surface area) might have enhanced their attachment to bacterial cellular membranes, permeability, and interaction ability with the bacterial internal components, including its DNA, resulting in superior bactericidal impact while interfering with vital functions necessary for bacterial survival.^1,2^ Future research should focus on the long-term effects of these bio-synthesized nanoparticles with more in-depth analysis of the mechanisms underlying the AgNPs activity.

### 3.4. Metallic nanoparticles synthesized using *Pistacia chinensis* extracts

Alhumaydhi (2022) reported the green synthesis of AuNPs using a *Pistacia chinensis* seed extract.^71^ The AuNPs were synthesized using a methanolic extract of *P. chinensis* seeds. One kilogram of shade-dried seeds was soaked in methanol for 10 days and then concentrated by rotary evaporation to yield 18.9 g of extract. For synthesis, 2 mg of extract was dissolved in 100 mL distilled water. This extract was mixed with 1 mM HAuCl_4_ solution at different volume ratios (1 : 1 to 1 : 12) and stirred at 40 °C for 30 min, followed by continuous stirring for 5 h. Color change indicated AuNPs formation. UV-Vis spectroscopy showed strong SPR peaks between 530 and 550 nm, with the 1 : 4 extract-to-HAuCl_4_ solution ratio giving the highest and sharpest peak, indicating optimal formation of uniform AuNPs.^71^ Atomic Force Microscopy (AFM) revealed AuNPs with a spherical shape and a size range of 10 to 100 nm.

The prepared AuNPs showed promising biological activities in treating enzyme-related disorders, pain, and anxiety. Both *P. chinensis* extract and AuNPs inhibited urease and carbonic anhydrase, with AuNPs showing superior activity (IC_50_ of 44.98 and 53.54 μg mL^−1^ against urease and carbonic anhydrase, respectively). Additionally, AuNPs depicted significant pain-relieving and sedative effects in animal models.^71^ The analgesic activity of both the *P. chinensis* extract and AuNPs was examined using an acetic acid-induced writhing model. AuNPs showed a promising analgesic effect. At the lowest dose tested (5 mg kg^−1^), the writhing number was 58.09 in 10 min (compared to the control group which showed 146.98 writhing). Notably, this level of pain relief was achieved at a significantly low dose compared to diclofenac sodium, the standard pain medication (83 writhing at 10 mg kg^−1^), and the *P. chinensis* extract (45.76 writhing at 100 mg kg^−1^). These findings might be interpreted by the effective process of synthesizing AuNPs from the extract, which might have concentrated the pain-relieving components, potentially leading to a more potent analgesic effect at lower doses.^71^ Nevertheless, an open-field test was conducted to investigate the sedative effects of AuNPs compared to the *P. chinensis* extract. Notably, at a dose of 15 mg kg^−1^, AuNPs exhibited a promising sedative activity (35.54 lines crossed) compared to the *P. chinensis* extract itself, where all doses (25, 50, and 100 mg kg^−1^) resulted in a lower number of lines crossed (60.98, 51.92, and 42.03, respectively).^71^ These results denote the promise of the synthesized AuNPs in various fields, whereas further studies are needed to reveal the underlying mechanisms and optimize the synthesis process.

### 3.5. Metallic nanoparticles synthesized using *Pistacia integerrima* extracts

*Pistacia integerrima*, also known as Karkatshringi, belongs to a medium-sized tree native to the Himalayan foothills. Traditionally, *P. integerrima* was exploited for various ailments such as skin inflammation, fever, and diarrhea disorders and was effectively investigated for its pain relief, radical-scavenging, and anti-inflammatory effects. The plant contains monoterpenes and flavonoids, among others, potentially contributing to its medicinal effects.^72^

Islam *et al.* (2019) successfully synthesized AuNPs utilizing the gall extract of *P. integerrima* as a reducing and stabilizing agent.^72^ The nanoparticles were synthesized using a methanolic extract of *Pistacia integerrima* galls. The galls were shade-dried, crushed, and extracted in methanol at room temperature for 7 days. The extract was concentrated under reduced pressure at 50 °C. For AuNPs formation, 2 g of the extract was dissolved in 100 mL absolute ethanol and mixed with varying volumes of 1 mM HAuCl_4_ solution. Bioreduction was monitored *via* UV-Vis spectroscopy (200–900 nm), and the nanoparticles were characterized by SEM and FTIR. Centrifugation at 10 000 rpm for 15 min and vacuum drying were performed to purify the samples prior to analysis.^72^ Hence, AuNPs formation was confirmed by the color change observed in UV-Vis analysis with a peak at 540 nm. The size of the synthesized AuNPs was determined by SEM analysis, showing a size range of 20 to 200 nm. Additionally, the FTIR spectra could further suggest the involvement of amines, amides, and alcohols in reduction and capping.^72^ Interestingly, the AuNPs remained stable over a wide range of pH values (2–12), with slight variations, whereas the nanoparticles showed moderate stability with increasing NaCl concentration (0.1 M). Also, AuNPs stability could further be evaluated under high temperatures, showing good thermal stability up to 80 °C for 30 min.^72^

**Fig. 11:**
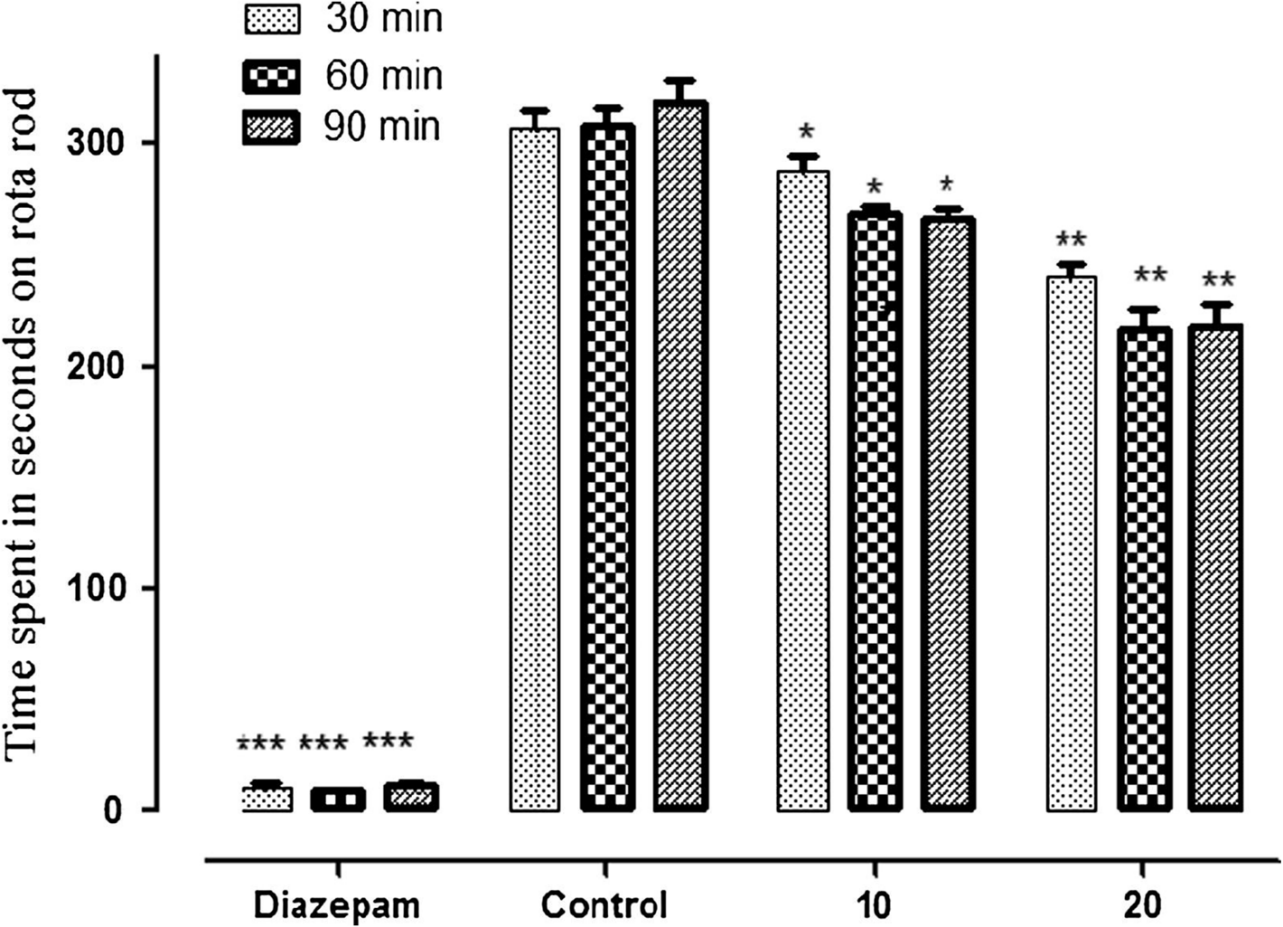
Muscle relaxant activity following treatment with specific doses of green-synthesized AuNPs (10 mg kg^−1^ and 20 mg kg^−1^). The treatment depicted a significant reduction in the time spent, in seconds, on the rotarod (represented by the bars shown above) compared to saline, following three specific treatment intervals of 30, 60, and 90 min.^72^ Reprinted from ref. 72. Copyright Elsevier 2019.

The *P. integerrima* extract exhibited a strong enzyme inhibition effect against carbonic anhydrase, xanthine oxidase, and urease with IC_50_ values of 23.45 μg mL^−1^, 21.45 μg mL^−1^, and 96.3 μg mL^−1^, respectively. However, the AuNPs lacked enzyme inhibitory activity against these enzymes. This might indicate that the active components responsible for enzyme inhibition in the extract (at concentrations below 96.3 μg mL^−1^) might not be directly translated or incorporated onto the nanoparticles. Interestingly, the AuNPs exhibited other promising activities. They depicted moderate antifungal activity against *A. niger* (20 ± 0.29 mm), *A. flavus* (10 ± 0.39 mm), and *Alternaria solani* (20 ± 0.27 mm) zones of inhibition, while the extract itself lacked such effects.^72^ Furthermore, AuNPs exhibited a considerable antinociceptive effect in the acetic acid-induced writhing test, with a maximum writhing inhibition of 80.76% observed at a dose of 20 mg kg^−1^, comparable to diclofenac sodium (82.54%). Additionally, AuNPs showed muscle relaxant properties at specific doses, with the time spent on the rotarod remarkably reduced following AuNPs treatment at 10 mg kg^−1^ and 20 mg kg^−1^ compared to saline (Fig. 11).^72^ These findings support potential utilization of AuNPs in several biomedical applications owing to their well-established stability, antifungal activity, and pain-relieving properties, and further research might be encouraged to reveal their potential in drug delivery and targeted therapies.

### 3.6. Metallic nanoparticles synthesized using *Pistacia terebinthus* extracts

Hailing from the Mediterranean region, the terebinth tree (*Pistacia terebinthus*), also recognized as the turpentine tree, is a shrub native to areas spreading from western Morocco and Portugal all the way to Greece and southeastern Turkey.^73–75^ Studies showed that *P. terebinthus* and its extracts possess potent antibacterial properties against several bacterial strains.^74,75^

**Fig. 12:**
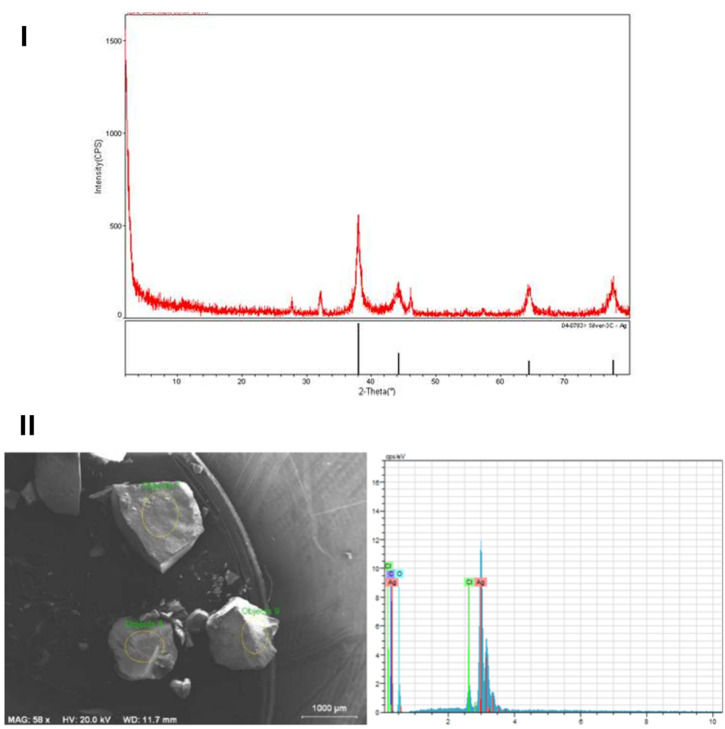
XRD (I) and SEM-EDX (II) spectra of green-synthesized AgNPs. While SEM-EDX analysis showed pure Ag phases, the XRD spectrum confirmed the face-centered cubic structure of the developed AgNPs with peaks at specific 2*θ* values (38.12°, 44.98°, 64.53°, and 77.32°) and also revealed a mean particle size of 15.62 nm.^73^ Reprinted from ref. 73. Copyright EJONS 2018.

Baran (2018) reported a green synthesis approach for AgNPs fabrication exploiting the leaf extract of *P. terebinthus* as a stabilizing and reducing agent.^73^ Nanoparticles were synthesized using an aqueous extract of *P. terebinthus* leaves. Fresh leaves were washed, shade-dried, and ground. 25 g of dried leaf powder was boiled in 500 mL of deionized water, cooled, filtered (Whatman No. 1), and stored at 4 °C. For AgNPs synthesis, 50 mL of this extract was mixed with 500 mL of 1 mM AgNO_3_ solution in a 1000 mL beaker at room temperature (25 °C). The reaction mixture gradually turned dark brown, indicating AgNPs formation. Completion of the reaction was monitored using UV-Vis spectroscopy by detecting the SPR peak maximum at 425.4 nm.^73^ Additionally, the obtained AgNPs were characterized by various techniques, including SEM, XRD, TGA, and FTIR. FTIR spectroscopy could confirm the presence of several functional groups of alkynyl, hydroxyl, and carboxyl groups, obtained from the extract, on the AgNPs surface, suggesting their role in stabilization.^73^ TGA analysis revealed high thermal stability of the AgNPs. The AgNPs exhibited minimal weight loss up to 180 °C. Further heating resulted in several stages of decomposition initiated by the degradation of the organic materials from the plant extract (7.02% weight loss between 180 °C and 309 °C), followed by dehydration of water molecules (11.37% weight loss between 309 and 610 °C), potentially associated with silver oxide decomposition. Finally, the significant weight loss (17.13%) between 610 °C and 900 °C refers to the degradation of the AgNPs themselves. Additionally, SEM analysis revealed spherical shapes of AgNPs with a mean size between 13 and 250 nm.^73^ While SEM-EDX analysis showed pure Ag phases, XRD confirmed the face-centered cubic structure of AgNPs with peaks at specific 2*θ* values (38.12°, 44.98°, 64.53°, and 77.32°) and revealed also a mean particle size of the nanoparticles of 15.62 nm (Fig. 12I and II).^73^

The antimicrobial efficacy of the AgNPs was assessed against *S. aureus*, *C. albicans* (fungus), and *E. coli*. The AgNPs exhibited substantial antibacterial and antifungal activity against all tested strains, with MIC values of 0.0203 mg mL^−1^ (*C. albicans*), 0.0812 mg mL^−1^ (*S. aureus*), and 0.325 mg mL^−1^ (*E. coli*). Notably, the AgNPs had more pronounced antimicrobial activity than AgNO_3_ solution.^73^ These findings suggest the potential of *P. terebinthus* leaf extract-mediated AgNPs for combating various microbial infections. Further research is required to ensure the long-term safety, efficacy, and environmental compatibility of these AgNPs.

**Fig. 13:**
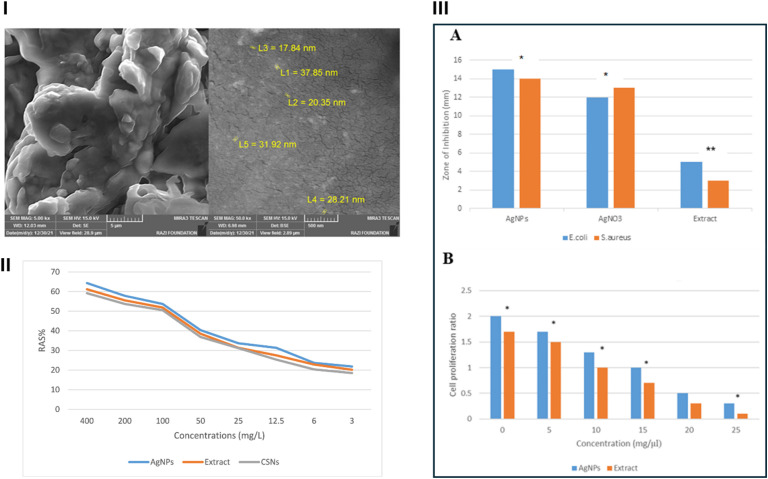
(I) SEM images of green-synthesized AgNPs at different magnifications, depicting a spherical shape and a mean size of 20–30 nm of the developed nanoparticles; (II) DPPH antioxidant assay of green-synthesized AgNPs over a range of concentrations (3 to 400 μg mL^−1^); (III) (A) antimicrobial properties investigation of green-synthesized AgNPs, AgNO_3_, and the free extract of *P. terebinth* employing an agar well diffusion test against *S. aureus* and *E. coli* bacteria, (B) anticancer property assessment of the developed AgNPs compared to the free extract against breast MCF-7 cancer cells.^74^ RAS: radical scavenging activity. Reprinted and modified with permission from ref. 74. Copyright Elsevier 2022.

Naghmachi *et al.* (2022) investigated a green synthesis approach for AgNPs fabrication employing a *P. terebinthus* extract, as a reducing and stabilizing agent, while exploring their therapeutic potential.^74^ The nanoparticles were synthesized using a methanolic extract of *P. terebinthus*. Leaf powder (10 g) was macerated in 100 mL methanol for 24 h and then filtered. For AgNPs synthesis, 50 g of the dried methanolic extract was mixed with 100 mL deionized water (pH adjusted to 8), and this extract was added to 1 mM AgNO_3_ solution at a 1 : 10 ratio. The reaction mixture was shaken at 150 rpm and kept at 37–40 °C in the dark for 24 h. The mixture's color changed from light brown to colloidal brown, indicating nanoparticles formation. AgNPs were purified by centrifugation at 12 000 rpm for 10 min (repeated 3 times), lyophilized, and stored. UV-Vis spectroscopy confirmed AgNPs formation with an SPR peak at 430 nm.^74^ While FTIR spectroscopy confirmed the chemical structure of AgNPs, XRD analysis could further confirm the crystalline structures of AgNPs with peaks at 2*θ* = 38.18°, 44.18°, 64.45°, and 77.30°. SEM analysis revealed spherical AgNPs with a mean size of 20–30 nm (Fig. 13I).^74^

The free radical-scavenging effect of AgNPs was evaluated by DPPH antioxidant assay, exhibiting a promising effect with a minimum concentration of 3 μg mL^−1^ (21.83%). The antioxidant activity increased to 64.33% upon increasing the concentration of AgNPs to 400 μg mL^−1^ (Fig. 13II).^74^ Furthermore, the antimicrobial properties of AgNPs were investigated employing agar well diffusion (Fig. 13IIIA). The AgNPs exhibited inhibition zones of 15 mm and 14 mm against *E. coli* and *S. aureus* bacteria, respectively, compared to the plant extract, which showed 5 mm and 3 mm zones of inhibition, respectively.^74^ Moreover, both AgNPs and *P. terebinthus* extract demonstrated cytotoxic effects on MCF-7 breast cancer cells. This effect was concentration-dependent, with cell viability decreasing as the concentration of both substances increased from 0 mg μL^−1^ to 25 mg μL^−1^. Hence, the highest cytotoxic effect was observed at the 25 mg μL^−1^ concentration for both treatments (Fig. 13IIIB).^74^ Though this study presents promising green-synthesized AgNPs with diverse bioactivities, further *in vivo* testing and exploration of action mechanisms are warranted. Table 1 summarizes methods used for *Pistacia*-mediated synthesis of metallic nanoparticles as well as their physiochemical characteristics and biological activities.

**Table 1:** *Pistacia*-mediated synthesis of metallic nanoparticles and their characteristics

*Pistacia* species (country)	Metallic nanoparticles prepared and synthesis method	Metallic nanoparticle characteristics
*Pistacia atlantica* (Iran)	AgNPs^60^	AgNPs (spherical; 10–50 nm) showed high stability at pH 11 (zeta potential −64.3 mV)
	2 g *P. atlantica* leaf powder was extracted in 25 mL water + 2 mL methanol. 1 mL extract was added to 10 mL of 1 mM AgNO_3_ and shaken at room temperature for 35 min. AgNPs were purified by centrifugation (10 000 rpm, 15 min) and dried at 60 °C. *P. atlantica* acted as both a reducing and capping agent	Antibacterial study showed superior activity against *S. aureus* compared to the extract (as shown by SEM only)
*Pistacia atlantica* (Iran)	PdNPs^61^	Synthesized highly monodisperse PdNPs (< 15 nm; zeta potential −24.5 mV) using a fruit extract
	PdCl_2_ (1 mM, dissolved in 0.1 M HCl) was mixed with *P. atlantica* fruit broth (15 mL extract: 100 mL PdCl_2_) and incubated at 85 °C. Pd(ii) reduced by phenolics/triterpenoids; starch acted as the stabilizer. pH dropped from 3.0 to 1.6, confirming bioreduction	FTIR suggested reduction of Pd(ii) ions by phenolics and triterpenoids, alongside fatty acids for stabilizing the PdNPs
		No biological or stability assays were conducted
*Pistacia atlantica* (Libya)	AgNPs^62^	AgNPs (7–17 nm) showed stable zeta potential (−10.6 to −23.6 mV)
	0.017 g AgNO_3_ in 100 mL H_2_O (1 mM); 1 mL *P. atlantica* leaf extract was added to 50 mL AgNO_3_ and incubated at room temperature in the dark. Ag^+^ reduced to AgNPs (7–17 nm); color changed to brown. NPs were centrifuged and vacuum dried	FTIR suggested phenolic/flavonoid involvement (gallic acid, hydrolysable tannins, ellagitannins, quercetin, luteolin, apigenin, kaempferol, galloyl and digalloyl glucosides)
		*In vivo* study demonstrated superior hepatoprotective effects *vs.* the free extract (improved ALT, AST, and ALP levels, liver histology, and antioxidant and anti-inflammatory properties)
*Pistacia atlantica* (Iran)	AuNPs^63^	AuNPs (spherical; TEM: 2.07–2.38 nm; SEM: 30–50 nm)
	10 mL extract (leaf/fruit/mix) + 100 mL of 1 mM HAuCl_4_ was stirred at room temperature; color change within 10–60 s (red-purple). Centrifuged at 12 000 rpm, washed, and dried at 50 °C. Particle size varied: leaf (30 nm), mix (40 nm), and fruit (50 nm). Extracts acted as reducing and stabilizing agents	HPLC confirmed the presence of phenolics (*e.g.*, gallic acid, rutin, and 4-hydroxybenzoic acid), which acted as reducing and capping agents
		Antioxidant: dose-dependent scavenging activity shown, reaching ∼45% at 80 μg mL^−1^ (DPPH), as compared to butylated hydroxytoluene that showed around 20% scavenging activity at the same concentration
		Strong antimicrobial activities against *B. subtilis, S. aureus*, *P. aeruginosa*, and *E. coli*. MICs: *P. aeruginosa* (3.9 μg mL^−1^), *S. aureus* & *E. coli* (7.81 μg mL^−1^), and *B. subtilis* (31.25 μg mL^−1^). MBCs: *S. aureus* & *E. coli* (15.62 μg mL^−1^) and *P. aeruginosa* & *B. subtilis* (62.5 μg mL^−1^). Inhibition zones reached 20–23 mm at 31 μg mL; ∼8 mm even at 3 μg mL^−1^
*Pistacia atlantica* (Iran)	AgNPs^64^	AgNPs showed promising antioxidant activity (IC_50_ = 0.47 mg mL^−1^)
	2.5 mL methanolic *P. atlantica* leaf extract + 1 mM AgNO_3_, incubated at 60 °C for 1 h (in the dark), cooled for 24 h, centrifuged (3600 rpm, 30 min), and dried at 90 °C. SPR peak at 468 nm confirmed AgNPs	Excellent antimicrobial properties against *Acinetobacter baumannii*, *S. aureus*, and *E. coli* (inhibition zones of 11 mm, 12 mm, and 13 mm, respectively)
*Pistacia atlantica* (Iraq)	AgNPs^65^	AgNPs (17–18 nm) showed strong antibacterial activity: zone of inhibition ranged from 6 mm (*S. pyogenes* and *K. pneumoniae*) to 25 mm (*P. aeruginosa*), with diameters of 7 mm (*S. paratyphi B*) and 8 mm (*S. aureus* and *E. coli*) observed
	100 g *P. atlantica* leaves were boiled in 500 mL water (30 min); 50 mL extract was added dropwise to 50 mL of 0.003 mL per L AgNO_3_ with stirring at 80 °C. Color change to yellowish brown indicated AgNPs formation *via* SPR. Extract served as a reducing and capping agent	
*Pistacia atlantica* (Iran)	AgNPs, ZnONPs, and AgZnO nanocomposites^66^	AgNPs, ZnONPs, and AgZnONPs (spherical; 30–60 nm) showed enhanced antibacterial activity *vs.* nanoparticles alone. AgNPs (12.5–15 mm) > AgNPs alone (10–15 mm); ZnONPs (13.6–17.5 mm) > ZnONPs alone (11.3–13.5 mm). AgZnONPs (resin-mediated) showed the strongest inhibition: *P. mirabilis* 21 ± 0.002 mm, *E. coli* 20 ± 1.2 mm, *S. aureus* 16 ± 0.3 mm, and *K. pneumoniae* 15 ± 0.08 mm *vs.* non-resin AgZnONPs (13–15.2 mm range). Resin extract alone had an inhibition zone of 7–11 mm
	Resin extract was dissolved in 10% ethanol/DMSO. AgNPs: 20 mL resin extract + 1 mL 0.001 M AgNO_3_, incubated at 70 °C for 5 min. ZnONPs: 3 mL resin extract + 47 mL 0.05 mM Zn(NO_3_)_2_ at 70 °C, and 150 min. AgZnONPs: 5 mL resin extract + 10 mL Zn(NO_3_)_2_ (0.05 M) + 5 mL AgNO_3_ (0.01 M), 70 °C, and 60 min	
*Pistacia atlantica* (Iran)	A nanocomposite incorporating AgNPs on a reduced graphene-oxide (RGO) sheet^67^	Phytochemical analysis suggested the presence of flavonoids, phenolic glycosides, tannins, coumarins, and saponins in the extract
	The nanocomposite was synthesized as follows: *P. atlantica* leaf extract (10 g/300 mL) was boiled for 30 min, added to 100 mg GO (100 mL H_2_O), sonicated for 30 min, and refluxed for 5 h. After washing, 20 mg AgNO_3_ in 20 mL was added and refluxed at 100 °C for 2 h. Final Ag content = 0.23 mmol g^−1^. Flavonoids, tannins, saponins, and phenolics facilitated reduction/stabilization	RGO–AgNPs showed strong, dose-dependent antibacterial activity. Max inhibition zone: *S. saprophyticus* (64.2 ± 1.3 mm at 64 mg mL^−1^). MICs: *S. pyogenes*, *B. subtilis*, *S. saprophyticus*, *P. aeruginosa* (1 mg mL^−1^); *S. aureus*, *E. coli* (2 mg mL^−1^); *S. typhimurium* (4 mg mL^−1^); *P. mirabilis* (8 mg mL^−1^). MBCs ranged from 1–8 mg mL^−1^. Extract alone showed minimal/no inhibition
*Pistacia atlantica* (Iran)	AgNPs^68^	AgNPs (spherical; 40–50 nm) showed 87.71% DPPH radical scavenging at 100 μg mL^−1^ (*vs.* 25.32% for BHT)
	100 g *P. atlantica* leaves were extracted in 1000 mL water (24 h) and concentrated at 40 °C. 2 g extract in 20 mL water; 2.5 mL was added to AgNO_3_ (5 × 10^−4^ M) and heated at 80 °C for 24 h. Color changed to black after 48 h. SPR peak at 440 nm confirmed AgNPs; formation influenced by pH (5–10) and extract/AgNO_3_ ratios	TGA showed thermal stability (total weight loss was 38.85% between 20–800 °C)
		HUVEC cell viability > 80% at 200 μg mL^−1^
		Antibacterial activity: *P. aeruginosa* MIC 1 μg mL^−1^, MBC 3 μg mL; *E. coli* MIC/MBC 3 μg mL; *S. aureus* and *B. subtilis* MIC 7 μg mL; MBCs 15 μg mL^−1^ (*S. aureus*), 7 μg mL^−1^ (*B. subtilis*). Inhibition zones visible at ≥31 μg mL^−1^
*Pistacia lentiscus* (Egypt)	AgNPs^70^	AgNPs (spherical; 24–26 nm) showed antibacterial activity (disc diffusion, mm): *P. aeruginosa*, *B. subtilis* (14 mm); *E. coli*, *S. aureus*, and *S. faecalis* (13 mm); *N. gonorrhoeae* (12 mm). Antifungal: Active *vs. A. flavus* (11 mm), inactive *vs. A. niger*
	5 g *P. lentiscus* leaves were boiled in 200 mL 50% EtOH (15–20 min) and filtered. 30 mL extract + 30 mL 0.025 M AgNO_3_ was incubated at 50 °C until color change	
*Pistacia vera* (Iran)	Copper nanoparticles (CuNPs) and CuNPs/silver Schiff base nanocomposite^69^	HPLC and GC-MS revealed phenolics (gallic acid, protocatechuic acid, catechin, rutin, and quercetin) and several terpenoid compounds
	15 g *P. vera* hull was sonicated in 150 mL water (45 °C, 20 min), centrifuged (6000 rpm, 30 min), and filtered. 8 mL extract was added dropwise to 100 mL of 8 mM Cu(ii) acetate at 70 °C, stirred for 5 h, and washed with distilled water, ethanol, and *n*-hexane. The obtained mixture was then dried overnight at room temperature, followed by an additional drying for one hour at 65 °C	CuNPs (15–45 nm) exhibited broad-spectrum antimicrobial activity. MICs (CuNPs): *E. coli*, *A. niger* (3.125 μg mL^−1^); *C. albicans*, *P. aeruginosa* (6.25 μg mL^−1^); *B. cereus* (12.5 μg mL^−1^); *S. aureus* (25 μg mL^−1^). CSS nanocomposite showed enhanced potency: MICs as low as 1.56 μg mL^−1^. Effective against both Gram-positive/negative bacteria and fungi
*Pistacia chinensis* (Pakistan)	AuNPs^71^	AuNPs (spherical; 10–100 nm) inhibited urease (IC_50_ = 44.98 μg mL^−1^) and carbonic anhydrase (IC_50_ = 53.54 μg mL^−1^)
	1 kg *P. chinensis* seeds were extracted in methanol (10 days) and concentrated (18.9 g yield). 2 mg extract in 100 mL water was mixed with 1 mM HAuCl_4_ at various ratios (1 : 1 to 1 : 12) and stirred for 30 min at 40 °C, followed by continuous stirring for an additional 5 h. Optimal ratio 1 : 4; SPR peak at 530–550 nm confirmed uniform AuNPs	Analgesic effect (acetic acid model): 58.09 writhes at 5 mg kg^−1^ (*vs.* 146.98 control, 83 diclofenac at 10 mg kg^−1^, and 45.76 extract at 100 mg kg^−1^)
		Sedative effect (open field test): 35.54 lines at 15 mg kg^−1^ (*vs.* 60.98–42.03 for the extract at 25–100 mg kg^−1^). Demonstrated superior bioactivity *vs.* the extract and standard drugs at lower doses
*Pistacia integerrima* (Pakistan)	AuNPs^72^	AuNPs (20–200 nm) were stable over pH 2–12 and at high temperatures (80 °C)
	*P. integerrima* galls were extracted in methanol (7 days, room temperature) and concentrated at 50 °C. 2 g extract in 100 mL ethanol was mixed with varying volumes of 1 mM HAuCl_4_. AuNPs formation was monitored by UV-Vis (200–900 nm) and characterized by SEM and FTIR after centrifugation (10 000 rpm, 15 min) and vacuum drying	FTIR spectra revealed the involvement of amines, amides, and alcohols in reduction and capping of the obtained AuNPs
		AuNPs showed moderate antifungal activity. Zones of inhibition: *A. niger* & *A. solani* (20 ± 0.3 mm); *A. flavus* (10 ± 0.4 mm)
		Antinociceptive effect: 80.76% writhing inhibition at 20 mg kg^−1^ (*vs.* 82.54% diclofenac)
		Muscle relaxant effect observed at 10–20 mg kg^−1^ (rotarod test)
*Pistacia terebinthus* (Turkey)	AgNPs^73^	FTIR suggested phenolics, flavonoids, and tannins, and an effective role of different functional groups (carboxyl, hydroxyl, and alkyl groups) in reducing and stabilizing the AgNPs
	25 g *P. terebinthus* leaves were boiled in 500 mL water, filtered, and stored at 4 °C. 50 mL extract was mixed with 500 mL of 1 mM AgNO_3_ at 25 °C. Color changed to dark brown; AgNPs formation was confirmed by the UV-Vis peak	High thermal stability with insignificant weight loss up to 900 °C (TGA)
		AgNPs (spherical, 13–250 nm) showed strong antimicrobial activity: MICs – *C. albicans* (0.0203 mg mL^−1^), *S. aureus* (0.0812 mg mL^−1^), and *E. coli* (0.325 mg mL^−1^). Outperformed silver nitrate solution in all tested strains
*Pistacia terebinthus* (Iran)	AgNPs^74^	FTIR revealed different functional groups of phenolics and organic acids responsible for the reduction and stabilization of AgNPs
	10 g leaves were extracted in 100 mL methanol (24 h) and filtered. 50 g dried extract in 100 mL water (pH 8) + 1 mM AgNO_3_ (1 : 10 ratio), shaken at 37–40 °C for 24 h. Color change to chestnut confirmed AgNPs. Purified by centrifugation (12 000 rpm) and lyophilized. UV-Vis peak at 430 nm	AgNPs (spherical; 20–30 nm)
		Antioxidant activity (DPPH): 21.83% at 3 μg mL^−1^, rising to 64.33% at 400 μg mL^−1^
		Antibacterial zones (mm): *E. coli* (15) and *S. aureus* (14); extract alone resulted in limited inhibition zones of 5 mm and 3 mm, respectively
		Anticancer activity: reduced MCF-7 cell viability concentration-dependent manner

## 4. Mechanistic insights into the biological activities of *Pistacia* extracts and their metal nanoparticles

Several bioactive compounds were identified in *Pistacia* species, demonstrating their significant role in the synthesis of metallic nanoparticles and highlighting their potential as natural agents for medical applications. Studies have extensively documented the diverse pharmacological properties of *Pistacia*-derived nanoparticles, including anti-inflammatory, antioxidant, anticancer and antimicrobial activities.^6,56,76^ This section provides a comprehensive review and discussion on the pharmacological effects of metallic nanoparticles synthesized using *Pistacia* extracts.

### 4.1. Antimicrobial activity

The antimicrobial efficacy of metallic nanoparticles derives from their unique ability to interact with microbial cells through multiple mechanisms, which include direct disruption of the cell membrane, generation of reactive oxygen species (ROS), release of metal ions, and interference with intracellular components critical for bacterial metabolism.^77,78^ In addition, green synthesis approaches using plant extracts, which serve as reducing and capping agents, impart enhanced biological activity and stability to the nanoparticles.^79,80^

The monoterpenes (myrcene, limonene, and α-pinene), which are enriched in essential oils of the *Pistacia* genus, exhibit diverse and complementary antimicrobial mechanisms.^81–83^ Myrcene modulates membrane fluidity and may enhance cellular uptake.^84,85^ Limonene disrupts the microbial membrane through lipid bilayer integration, causing leakage of intracellular components and impairing energy metabolism, while also inhibiting biofilm formation.^86,87^ α-Pinene exerts bactericidal effects *via* membrane destabilization, inhibition of key microbial enzymes, and attenuation of bacterial resistance pathways.^88,89^ Together, these terpenes demonstrate significant potential as phytochemical-based antimicrobials, with the capacity to disrupt multiple microbial targets.

**Fig. 14:**
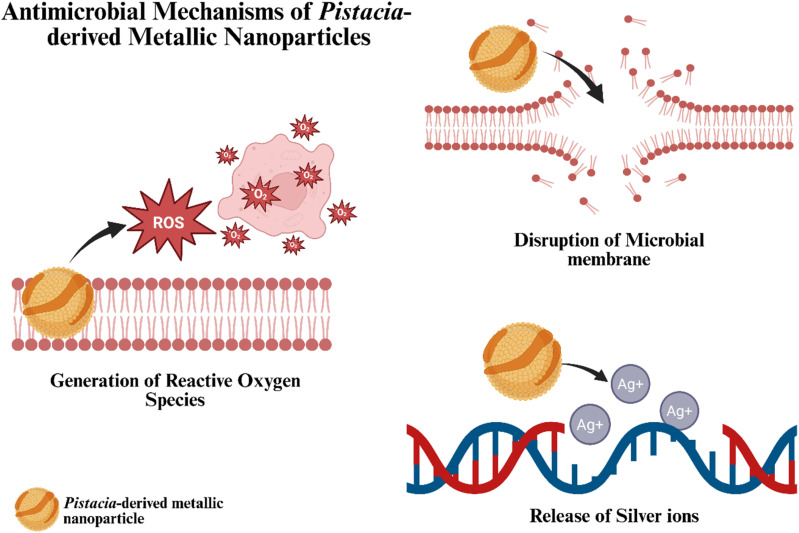
Schematic representation depicting the molecular mechanisms of *Pistacia*-derived metallic nanoparticles' antimicrobial activity including disrupting microbial cell membranes, generating reactive oxygen species (ROS), and releasing metal ions that interfere with vital cellular functions. The figure was drawn using BioRender.

Metallic nanoparticles prepared using *Pistacia* extracts demonstrated a robust antimicrobial effect by disrupting microbial cell membranes, generating ROS, and releasing metal ions that interfere with vital cellular functions (Fig. 14).^60,65,90,91^ Their enhanced biocompatibility and adaptability for composite formation further extend their potential applications in medicine and environmental remediation.^92,93^

AgNPs green synthesized using a *Pistacia atlantica* leaf extract disrupt bacterial membranes due to their nanoscale size and surface reactivity, leading to increased permeability and cell lysis. These nanoparticles also catalyze the generation of ROS, including superoxide and hydroxyl radicals, which damage cellular components and induce oxidative stress-mediated microbial death.^65,68,92,94^ Also, *Pistacia*-mediated AgNPs release silver ions (Ag^+^) that interfere with bacterial enzymes and DNA replication, enhancing bactericidal efficacy while reducing the risk of resistance development. On the other hand, AuNPs synthesized using a *Pistacia integerrima* gall extract have also demonstrated antimicrobial potential, possibly through membrane disruption and protein interference, with enhanced activity when functionalized with phytochemicals.^72,92,94^ Moreover, a *Pistacia vera* hull extract has been employed in the biosynthesis of hybrid nanocomposites, such as copper-silver Schiff base complexes, which exhibited superior antimicrobial performance and physicochemical stability compared to single-metal systems.^69,92,94^

### 4.2. Anticancer activity

Metallic nanoparticles exert anticancer effects through multiple interrelated mechanisms. A primary mode of action involves the generation of ROS, which induce oxidative stress within malignant cells, leading to mitochondrial dysfunction, DNA fragmentation, and activation of apoptosis. Apoptotic induction occurs *via* both intrinsic and extrinsic pathways: the intrinsic pathway is characterized by mitochondrial membrane depolarization, cytochrome C release, and activation of caspases such as caspase-3, while the extrinsic pathway is initiated through ligand-mediated activation of receptors, responsible for apoptosis leading to cancer cell death. Additionally, certain metallic nanoparticles contribute to tumor suppression through localizing the hyperthermic effect into cancer cells, augmenting susceptibility to cytotoxic damage.^95,96^

Essential oils derived from *Pistacia atlantica* are notably enriched with monoterpenes such as myrcene, limonene and α-pinene.^97^ The anticancer efficacy of these bioactive compounds is driven by their capacity to induce mitochondrial dysfunction, generate oxidative stress, activate caspase-dependent apoptosis, and arrest cell cycle progression in malignant cells.^98–101^ Myrcene's primary mode of action is through the induction of ROS-mediated apoptosis and cell cycle inhibition.^99,102^ Limonene exerts broad anticancer effects *via* mechanisms that include inhibition of tumor initiation, growth, and angiogenesis and induction of cancer cell apoptosis.^100^ Similarly, α-pinene demonstrates dual functionality by triggering apoptosis through mitochondrial damage and ROS accumulation.^101^

In breast cancer models, extracts from *Pistacia vera* hulls exhibited cytotoxic and anti-angiogenic effects against MCF-7 cells, suggesting their potential to inhibit tumor vascularization and proliferation. Additionally, extracts of *Pistacia atlantica* induced apoptosis and S-phase arrest in colon carcinoma HT29 cells, further supporting the role of *Pistacia*-derived phytochemicals in modulating cell cycle progression and apoptotic signaling.^103,104^

CuNPs synthesized from the *Pistacia khinjuk* leaf extract demonstrated significant cytotoxicity against prostate cancer cells by inducing mitochondrial apoptosis. This was mediated through tubulin polymerization inhibition and cytoskeletal disruption, leading to mitochondrial membrane depolarization and cytochrome C release, which activated caspase-dependent apoptosis.^105–107^

Similarly, AgNPs synthesized using the *Pistacia atlantica* bark extract showed strong anticancer effects against gastric cancer cells. These nanoparticles elevated intracellular ROS levels, caused DNA fragmentation, and arrested the cell cycle in the S phase, unlike conventional chemotherapeutics, such as doxorubicin, which induces G2/M arrest.^103,104,107^

### 4.3. Antioxidant and anti-inflammatory activities

Metallic nanoparticles are known to mimic the activity of natural antioxidant enzymes, such as superoxide dismutase, catalase, and peroxidases, by catalyzing the conversion of reactive oxygen species into less harmful molecules like water and oxygen.^108–112^ On the other hand, ZnONPs were reported to exert anti-inflammatory effects through blocking pro-inflammatory cytokines such as IL-1, IL-1β, and TNF-α, inhibiting the mast cell proliferation, and suppressing the expression of iNOS.^112–117^

Myrcene and α-pinene, which are enriched in essential oils of *Pistacia* genus exhibit unique and overlapping antioxidant mechanisms. Myrcene is reported to downregulate pro-inflammatory cytokines (IL-1β, IL-6, and TNF-α), immunomodulatory factors (IFNγ and NF-κB), and anti-inflammatory markers (IL-4 and IL-10).^118^ α-Pinene is shown to inhibit the expression of the inflammatory mediators of nuclear factor-kappa B (NF-κB), TNF-α and IL-6.^88^

AgNPs synthesized using the *Pistacia atlantica* bark extract were demonstrated to exert significant antioxidative and anti-inflammatory effects through activation of the Nrf2/ARE signaling axis, leading to upregulation of key antioxidant enzymes including heme oxygenase-1, glutathione peroxidase, and superoxide dismutase. This activation restores intracellular glutathione levels, reduces lipid peroxidation, and attenuates nitric oxide accumulation, thereby enhancing cellular redox homeostasis.^62,107,119^ Moreover, they downregulate alpha-fetoprotein gene expression and modulate nitric oxide synthase activity, contributing to hepatocyte regeneration and protection against oxidative liver injury in thioacetamide-induced acute liver failure models.^62,90,94^ Additionally, *P. atlantica*-derived AgNPs prevented NF-κB nuclear translocation and subsequent transcription of pro-inflammatory cytokines such as TNF-α, IL-1β, and IL-6. This anti-inflammatory effect was evidenced by reduced cytokine expression, decreased inflammatory cell infiltration, and improved histological architecture in both hepatic and gastric tissue models.^60,62,107,120^

## 5. Biosafety

Utilizing *Pistacia* species extracts in the synthesis of metallic nanoparticles is a promising approach for enhancing biocompatibility while minimizing potential toxicity. The capping agents provided by *Pistacia* extracts influence the physicochemical properties, stability, and biological interactions of the nanoparticles.^63,121,122^ AgNPs synthesized from an aqueous leaf extract of *Pistacia atlantica* were non-toxic in the concentration ranges tested.^68^*Pistacia*-derived AgNPs successfully ameliorated thioacetamide (TAA)-induced acute liver failure. It led to significant improvements in liver function biomarkers, lipid profiles, and oxidative stress indices without inducing any systemic toxicity. Histopathological examinations confirmed the absence of inflammatory or degenerative changes in hepatic and other tissues.^62^ CuNPs synthesized *via* the *P. atlantica* leaf extract exhibited protective effects in diabetic rats, improving glucose regulation, cardiac function, and liver enzyme levels without significant toxicological consequences. Oral administration up to 125 mg kg^−1^ over 28 days showed biochemical stability and no histopathological damage.^122^ While these results are promising, a comprehensive evaluation of genotoxicity, including assessments in multiple cell types and over extended exposure durations, is necessary to rule out genotoxic potential. Detailed pharmacokinetic and pharmacodynamic studies are needed to ensure that these nanoparticles do not accumulate in critical organs such as the liver, kidneys, heart, or lungs.^72,107,123,124^

## 6. Stability

**Fig. 15:**
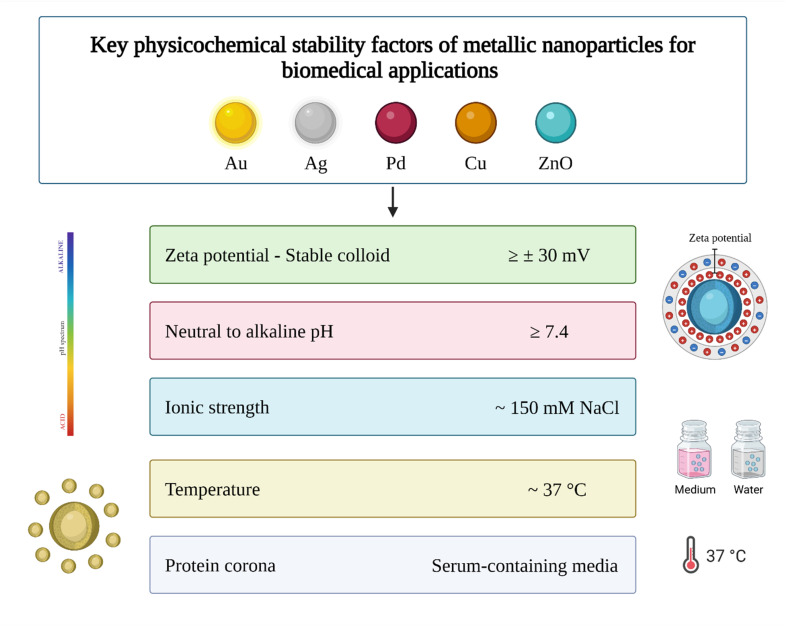
Key physicochemical parameters affecting the stability of metallic nanoparticles under physiological conditions. A zeta potential ≥ ±30 mV is generally associated with stable colloids. Ideal conditions include physiological pH (∼7.4), body temperature (37 °C), and ionic strength (commonly ∼150 mM NaCl). In biological media, protein adsorption leads to the formation of a “protein corona”, which can significantly impact nanoparticle stability, surface properties, and *in vivo* behavior. The figure was drawn using BioRender.

Investigating the stability of metallic nanoparticles under physiological conditions is necessary to support their biomedical application. Ideally, metallic nanoparticles should remain stable in biological media (pH ∼ 7.4, 37 °C, in presence of salts and other biomolecules), avoiding aggregation or surface charge neutralization. Zeta potential values exceeding ±30 mV are typically indicative of good colloidal stability and a reduced tendency to aggregate (Fig. 15).^125–127^

The surface charge of metallic nanoparticles promotes repulsion among particles, ensuring dispersion and enhancing their stability and biological interactions.^128^ The pH during synthesis also influences nanoparticle characteristics such as size, morphology, and stability. While neutral pH (∼7) often supports reduction of metal ions, alkaline pH (8–10) has been associated with improved nanoparticle formation in many green synthesis approaches.^129–131^

Ionic strength significantly impacts the colloidal stability and performance of metallic nanoparticles in various applications, including drug delivery and imaging. Nanoparticle aggregation, which directly influences cellular and tissue penetration and target interaction, is sensitive to ionic concentration.^132–135^ In low ionic strength environments (*e.g.*, deionized water or diluted salt solutions), electrostatic repulsion among similarly charged nanoparticles promotes dispersion. Conversely, high ionic strength conditions, such as those found in physiological fluids, can screen this repulsion, leading to aggregation. Therefore, an optimal ionic strength is crucial for maintaining colloidal stability while ensuring effective biological performance.^136,137^

Temperature plays a multifaceted role in the synthesis and application of metallic nanoparticles. For synthesis, temperatures typically range from 25 °C to 60 °C, though higher temperatures may be employed for accelerated reaction rates and processing. Lower temperatures are often preferred in specific applications to mitigate nanoparticle aggregation or degradation, thereby enhancing their long-term stability.^130,138–140^ For instance, in biomedical applications like cancer detection and treatment, nanoparticles are frequently utilized at or near body temperature (37 °C). Beyond stability, temperature also forms the basis for therapeutic approaches such as photothermal therapy, where nanoparticles are intentionally heated to ablate cancer cells.^130,138^

Upon entering physiological environments, metallic nanoparticles rapidly interact with biomolecules, especially serum proteins, forming a dynamic protein corona. This dynamic layer can alter the nanoparticle's size, surface charge, and biological identity. Depending on composition, the protein corona may stabilize particles through steric hindrance or promote aggregation, by altering surface charge and colloidal behavior. Understanding this interaction is vital for predicting their *in vivo* behavior.^141,142^

Sadeghi *et al.* demonstrated high stability over a wide range of pH (7 to 11) coupled with a remarkable zeta potential, reaching −64.3 mV, for *P. atlantica*-derived AgNPs, while Islam *et al.* showed that *P. integerrima*-derived AuNPs remained stable across a broad pH range (2–12) and at elevated temperatures (up to 80 °C for 30 min).^60,72^ Additionally, TGA analyses in several studies confirmed the thermal robustness of these nanoparticles. However, most studies that utilized *Pistacia* extracts to prepare metallic nanoparticles did not evaluate stability under physiologically relevant conditions such as serum-containing media or simulated body fluids. Hence, assessing the long-term colloidal behavior, ionic strength tolerance, and protein corona effects of *Pistacia*-synthesized nanoparticles, as demonstrated in other nanoparticle systems, is warranted.^143–146^

## 7. Conclusions and future prospects

This review highlights the remarkable potential of using extracts from various *Pistacia* species for the green synthesis of metallic nanoparticles with enhanced physicochemical properties, including thermal stability (resistance to decomposition at elevated temperatures), chemical resistance (stability in acidic or alkaline environments and tolerance to salt concentrations), structural integrity and morphological characteristics as could be seen by FTIR, XRD, UV-Vis, AFM, EDX, SEM, and TEM analyses (preservation of functional groups and maintenance of crystalline structures), and optical properties (specific absorption peaks in UV-Vis spectra and tunable optical properties upon formation of related nanoparticles). These enhancements, coupled with the significant biological activities of the green synthesized metallic nanoparticles mediated by *Pistacia* species extracts, including remarkable antibacterial, antifungal, antioxidant, anticancer, anti-inflammatory effects, along with promising safety profiles against normal cell lines, make these extracts a promising and versatile approach for the synthesis of functional nanomaterials with diverse applications.

*Pistacia* extracts could be recognized as a valuable plant source with well-established medicinal properties, in which one of these species (*P. lentiscus*) has been officially approved as a natural medicine by the European Committee on Herbal Products (HMPC) (July 22, 2015), as outlined in the EU pharmacopeia. The extracts derived from *Pistacia* species have various health benefits, including antibacterial, pain-relieving, anti-inflammatory, and antiviral effects.

Hence, the review emphasized the advantages of green synthesis of metallic nanoparticles, including its eco-friendly nature, rapid processing, and the inherent remarkable bioactivity of *Pistacia* extracts. By exploring the diverse applications of these green-synthesized metallic nanoparticles in pharmaceutical and biomedical fields, this review aimed to contribute to the ongoing advancements in nanomedicine.

Notably, *Pistacia* extracts have demonstrated significant biological activities, particularly in the areas of antimicrobial and anticancer properties. For antimicrobial activity, eleven studies have reported significant antibacterial properties of green synthesized nanoparticles derived from *Pistacia* extracts against different bacterial strains, including *Escherichia coli*, *Staphylococcus aureus*, *Pseudomonas aeruginosa*, *Salmonella species*, and others. These extracts demonstrated comparable or superior antimicrobial activity compared to free extracts. Similarly, for antifungal activity, four studies have highlighted antifungal activity against several fungal strains, such as *Aspergillus niger*, *A. flavus*, *Candida albicans*, and *Alternaria solani*. On the other hand, for anticancer activity, two *in vitro* studies have investigated the cytotoxic properties of synthesized nanoparticles against breast cancer cells (MCF-7) and normal cells (HUVECs), demonstrating promising cytotoxic effects against cancer cells while preserving a safe profile against normal cells. Furthermore, one study investigated the *in vivo* hepatoprotective and anti-inflammatory potential of prepared nanoparticles against acute liver failure induced by thioacetamide, as compared to free extracts, showing mostly higher hepatoprotective effects with the groups treated with nanoparticles. For the other biological activities reported, five studies have reported the antioxidant properties of metallic nanoparticles synthesized utilizing *Pistacia* extracts, which can contribute to their potential health benefits, whereas one study reported significant sedative and analgesic effects *in vivo*, and one study reported substantial muscle relaxant and antinociceptive effects revealed by green synthesized metallic nanoparticles. Overall, the evidence from these studies suggests that *Pistacia* extracts are a valuable source of bioactive compounds benefiting this green synthesis approach for nanomaterial fabrication with potential applications in various fields, including medicine and pharmaceuticals.

The reported biological enhancements are attributed to multiple interrelated pathways, including microbial membrane disruption, ROS generation, the release of bioactive metal ions, the inhibition of significant proinflammatory and proapoptotic markers, *etc*. The presence of phytochemicals such as phenolics and flavonoids contributes to these effects by enabling both nanoparticles stabilization and interactions with biological systems.

The future of green synthesis of metallic nanoparticles using *Pistacia* extracts holds immense promise. Further research should focus on optimizing green synthesis protocols to better control the shape, surface, and size features of metallic nanoparticles, exploring other *Pistacia* species and extracts for metallic nanoparticle synthesis, and in-depth evaluation of biological activities with a focus on evaluating the antimicrobial, anticancer, and other potential biological activities of green-synthesized particles. Special attention should be given to illustrating mechanisms of biological activities and toxicity assessments. It is also important to note that most of the reviewed studies did not report synthesis efficiency or nanoparticle yield, which presents a limitation for comparative analysis and future scalability. Therefore, future research could address these gaps by including such parameters as standard reporting metrics.

Although a few studies have reported stability data (*e.g.*, favorable zeta potential at physiological pH), systematic evaluations of nanoparticle behavior under physiologically relevant conditions, including temperature, ionic strength, and presence of biomolecules, are generally lacking. Future studies should incorporate these assessments to ensure translational viability of *Pistacia*-derived nanoparticles. Additionally, the potential use of the green synthesized particles for targeted drug delivery and translating promising results to *in vivo* studies and eventually clinical trials for therapeutic applications could be addressed to reveal the full potential of sustainable synthesis of metallic nanoparticles using *Pistacia* extracts.

## Conflicts of interest

The authors declare no potential competing interests.

## Data Availability

No primary research results, software or code have been included, and no new data were generated or analyzed as part of this review.
